# Svalbard reindeer winter diets: Long‐term dietary shifts to graminoids in response to a changing climate

**DOI:** 10.1111/gcb.16420

**Published:** 2022-09-17

**Authors:** Tamara A. Hiltunen, Audun Stien, Maria Väisänen, Erik Ropstad, Jouni O. Aspi, Jeffery M. Welker

**Affiliations:** ^1^ Ecology and Genetics Research Unit University of Oulu Oulu Finland; ^2^ Department of Arctic and Marine Biology, Fram Centre The Arctic University of Norway Tromsø Norway; ^3^ Arctic Centre University of Lapland Rovaniemi Finland; ^4^ Department of Production Animal Clinical Sciences Norwegian University of Life Sciences Ås Norway; ^5^ UArctic Rovaniemi Finland; ^6^ Department of Biological Sciences University of Alaska Anchorage Anchorage Alaska USA

**Keywords:** Arctic, climate change, diet, graminoids, rain‐on‐snow, *Rangifer tarandus platyrhynchus*, stable isotope, Svalbard, Svalbard reindeer, trophic niche

## Abstract

Arctic ecosystems are changing dramatically with warmer and wetter conditions resulting in complex interactions between herbivores and their forage. We investigated how Svalbard reindeer (*Rangifer tarandus platyrhynchus*) modify their late winter diets in response to long‐term trends and interannual variation in forage availability and accessibility. By reconstructing their diets and foraging niches over a 17‐year period (1995–2012) using serum δ^13^C and δ^15^N values, we found strong support for a temporal increase in the proportions of graminoids in the diets with a concurrent decline in the contributions of mosses. This dietary shift corresponds with graminoid abundance increases in the region and was associated with increases in population density, warmer summer temperatures and more frequent rain‐on‐snow (ROS) in winter. In addition, the variance in isotopic niche positions, breadths, and overlaps also supported a temporal shift in the foraging niche and a dietary response to extreme ROS events. Our long‐term study highlights the mechanisms by which winter and summer climate changes cascade through vegetation shifts and herbivore population dynamics to alter the foraging niche of Svalbard reindeer. Although it has been anticipated that climate changes in the Svalbard region of the Arctic would be detrimental to this unique ungulate, our study suggests that environmental change is in a phase where conditions are improving for this subspecies at the northernmost edge of the *Rangifer* distribution.

## Graphical abstract

Climate change results in complex interactions between Arctic herbivores and their forage. We document a long‐term temporal shift in the late winter diet of Svalbard reindeer (*Rangifer tarandus platyrhynchus*) toward more grasses and less low‐quality mosses. It has been anticipated that climate changes in the Svalbard region of the Arctic would be detrimental to this unique ungulate. However, the increased utilization of higher‐quality winter forage, may have played a causal role in the recent unexpected rise in reindeer densities in our study population.

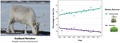

## Abstract (fi)

Tiivistelmä

Arktisella alueella laiduntavien eläinten ja kasvillisuuden väliset vuorovaikutukset muuttuvat nyt monitahoisesti, kun lämpötilat kohoavat ja kosteus lisääntyy. Tutkimme, kuinka pitkäaikaiset trendit sekä vuotuiset vaihtelut ravintokasvien saatavuudessa vaikuttavat huippuvuortenpeuran (Rangifer tarandus platyrhynchus) lopputalvella käyttämään ruokavalioon, ja ovatko nämä vaikutukset selitettävissä optimaalisen ravinnonhakuteorian avulla. Peurojen ravinto ja ravintoa vastaavat ekolokerot rekonstruoitiin 17 vuoden ajanjaksolta (1995–2012) mittaamalla peurojen seerumin hiilen ja typen isotooppiarvoja (δ^13^C, δ^15^N). Mittauksemme osoittivat vahvasti, että tutkimusjakson aikana heinäkasvien osuus kasvoi sammalten osuuden samanaikaisesti vähetessä peurojen ruokavaliossa. Havaittu ruokavalion muutos sopii hyvin yhteen tutkimusalueelta raportoituun heinäkasvien lisääntymiseen saman ajanjakson aikana. Eri isotooppien osuuksien muutokset olivat yhteydessä peuran populaatiotiheyden kasvuun, korkeampiin kesälämpötiloihin ja lumen päälle tulevien vesisateiden yleistymiseen. Näiden tekijöiden yhteisvaikutus johti heinäkasvien käytön lisääntymiseen mallinnetussa peuran talviruokavaliossa. Tämän lisäksi isotooppien avulla rekonstruoitujen ekolokeroiden asemoitumisessa, leveyksissä ja päällekkäisyyksissä havaittu vaihtelu tuki ravintoekolokeron ajallista muutosta erityisesti vasteena äärimmäisiin talvisiin vesisateisiin. Pitkäkestoinen tutkimuksemme havainnollisti, miten ilmaston‐ ja kasvipeitteen muutos sekä kasvanut populaatiotiheys voivat kumulatiivisesti vaikuttaa huippuvuortenpeuran ravinnonkäyttöön. Toisaalta havaintomme paljastavat myös, miten hienovaraisesti talvi‐ ja kesäilmaston muutos kumuloituvat kasvipeitteen ja laiduneläinten populaatiodynamiikan muutoksiksi. Löydöksiämme voidaan soveltaa luonnonsuojelussa, sillä niiden avulla voidaan torjua huippuvuortenpeuran sekä muiden arktisten laiduntajien populaatioromahduksia sekä ylläpitää arktisten ekosysteemien haurasta tasapainoa.

## 1 INTRODUCTION

Arctic ecosystems are changing in response to a warmer and wetter climate, leading to complex and varied responses by different species (Box et al., 2019; Chapin et al., 2008; Mallory & Boyce, 2018; Post & Stenseth, 1999). The warming and lengthening of the Arctic growing season has induced plant community shifts, increased primary production and in some cases increased the availability of palatable forage for herbivores (Elmendorf et al., 2012; Mallory et al., 2020; May et al., 2020; Myers‐Smith et al., 2020; Post et al., 2009). In contrast, winter warming may have adverse effects on forage availability. Winter precipitation at above‐zero temperatures may result in rain‐on‐snow (ROS) and ice layers that encapsulate vegetation (Andrews, 1996; Putkonen & Roe, 2003; Robinson et al., 1998). ROS events have resulted in winter starvation of ungulates, such as musk‐ox (*Ovibos moschatus*), semi‐domesticated and wild reindeer/caribou (*Rangifer tarandus*), and climate change is expected to make such events more frequent (Forbes et al., 2016; Garrott et al., 2003; Kumpula, 2001; Rennert et al., 2009). Although an increased frequency of ROS events has been predicted to destabilize the population dynamics of many ungulate species (Forbes et al., 2016; Hansen et al., 2010a; Pedersen et al., 2021; Putkonen & Roe, 2003), they have been shown to reduce extinction risk by stabilizing the population dynamics in Svalbard reindeer (*R. t. platyrhynchus*; Hansen, Gamelon, et al., 2019).

Svalbard reindeer population densities exhibit interannual variation caused by density‐dependent (i.e., competition for food resources) and density‐independent factors (i.e., climate; Hansen, Pedersen, et al., 2019; Pacyna et al., 2018). In the most productive parts of Svalbard, populations of Svalbard reindeer have increased since the turn of the century (Hansen, Gamelon, et al., 2019; Le Moullec et al., 2019). The positive population trends have been attributed to increased summer temperatures that have led to increased plant productivity and higher forage availability (Hansen et al., 2013; van der Wal & Stien, 2014; Vickers et al., 2016). Furthermore, a lengthening of the snow‐free autumn season allows reindeer easier access to food for longer periods of the year and thus gain more fat reserves before the winter (Albon et al., 2017; Loe et al., 2020). Severe winters have been shown to alter the population structure (demography), with elevated mortality in calves, males, and old‐age classes, and poor calf production the following summer (Albon et al., 2017; Peeters et al., 2017). However, the negative effects of difficult winters are reduced by behavioural responses to ROS, such as Svalbard reindeer migrating in search of nearby ice‐free foraging sites (Loe et al., 2016; Stien et al., 2010).

Climatic impacts on forage availability is one of the main drivers of Svalbard reindeer population dynamics (Albon et al., 2017). Optimal foraging theory (OFT; Pyke et al., 1977) predicts that generalist herbivores will shift from an optimal high‐quality diet to one that maximizes the quantity of forage when forage resources are reduced or inaccessible. Reindeer winter diets are typically dominated by energy‐rich fruticose lichens (Skogland, 1984), but on Svalbard lichens are scarce. Low‐quality, energy‐poor mosses contribute between 30% and 50% to the winter diets of Svalbard reindeer (Bjørkvoll et al., 2009; van der Wal, 2006; Węgrzyn et al., 2019). However, the low growing stature of mosses is likely to make them inaccessible when iced over during ROS events. Some studies have tested OFT using the winter diets of Svalbard reindeer and have captured a temporal “snapshot” of either the inter‐annual dietary variability over a couple of years or the direct response to a rare extreme event (Beumer et al., 2017; Bjørkvoll et al., 2009; Hansen & Aanes, 2012; Hansen, Lorentzen, et al., 2019). For instance, Bjørkvoll et al. (2009) concluded that during winters with ROS (2000–2002), Svalbard reindeer forage less on mosses and, instead, prefer plants with erect growth forms, such as graminoids, or plants growing in more exposed habitats, such as the dwarf shrubs, polar willow (*Salix polaris*) and mountain avens (*Dryas octopetala*). It is currently unclear what the long‐term patterns of Svalbard reindeer winter diets are, in response to the cumulative impacts of increased population density, increased frequency of ROS events, increased summer temperatures and potentially increased forage abundance.

Dietary information at different temporal scales can be effectively inferred using stable isotope analysis (SIA) of different tissues (Ben‐David & Flaherty, 2012; Fry, 2006; Rogers et al., 2015, 2020; Stanek et al., 2017, 2019). Progressive shifts in a herbivore's stable isotope (δ^13^C and δ^15^N) values reflect dietary changes as a result of differential tissue growth and turnover rates: from serum at 1–2 weeks → red blood cells at 2–3 months → muscle at 6–9 months → → bone over the lifetime (Dalerum & Angerbjörn, 2005; Libby et al., 1967; Tieszen et al., 1983). In addition to diet, intrinsic processes such as nutritional stress have variable effects on a herbivore's δ^13^C and δ^15^N values in different tissues (Barboza et al., 2020; Doi et al., 2017; Funck et al., 2020; Parker et al., 2005), and pregnancy may deplete serum δ^15^N in the 2 months preceding birth (Barboza & Parker, 2006). SIA was effectively used to verify that during extreme icing events coastal populations of Svalbard reindeer use marine forage (seaweed; Hansen, Lorentzen, et al., 2019). Thus, diets and dietary niches reconstructed from the variations in δ^13^C and δ^15^N may also indicate how inland populations of Svalbard reindeer are and may be capable of adapting their foraging strategy to interannual climate variation and climate change.

The overall objective of this study was to identify how Svalbard reindeer modify their winter diets in response to long‐term trends and interannual variation in forage availability over a 17‐year period (1995–2012). Svalbard reindeer are the only ungulate in a relatively simple food web where they are subjected to insignificant levels of interspecific competition and predation. This makes them an excellent study system to evaluate herbivore winter foraging ecology in the context of climate change (Hansen, Pedersen, et al., 2019). We collected serum from Svalbard reindeer in late winter, which is the critical period for their winter survival and reconstructed their diets using serum δ^13^C and δ^15^N values. Specifically, we asked whether Svalbard reindeer's late winter (1) serum isotopic values vary over time, (2) dietary composition shows temporal variation, and (3) isotopic niche spaces vary over time in terms of area and/or overlap. In addition to Bayesian stable isotope mixing and isotopic niche space models (Jackson et al., 2011; Parnell et al., 2010), we used linear regressions to explore (4) potential mechanisms that may drive trends in serum isotopic values. Furthermore, we evaluated how serum isotopic values were associated with variation in forage production (summer warming), forage accessibility (ROS), intra‐specific competition (population density), and intrinsic drivers like nutritional status (body mass) and status concerning pregnancy. We hypothesized, that graminoids and the deciduous shrub, *S. polaris*, would increase in the winter diet over time, as these plants were expected to increase in their cover in Svalbard (Hansen et al., 2010b; van der Wal & Brooker, 2004). Furthermore, we hypothesized that the proportion of mosses in the diet would decline when covered by ice due to ROS, while plants (i.e., graminoids) with erect growth forms growing in exposed habitats would often be accessible above ice layers and increase.

## 2 MATERIALS AND METHODS

### 2.1 Study area and species

**FIGURE 1:**
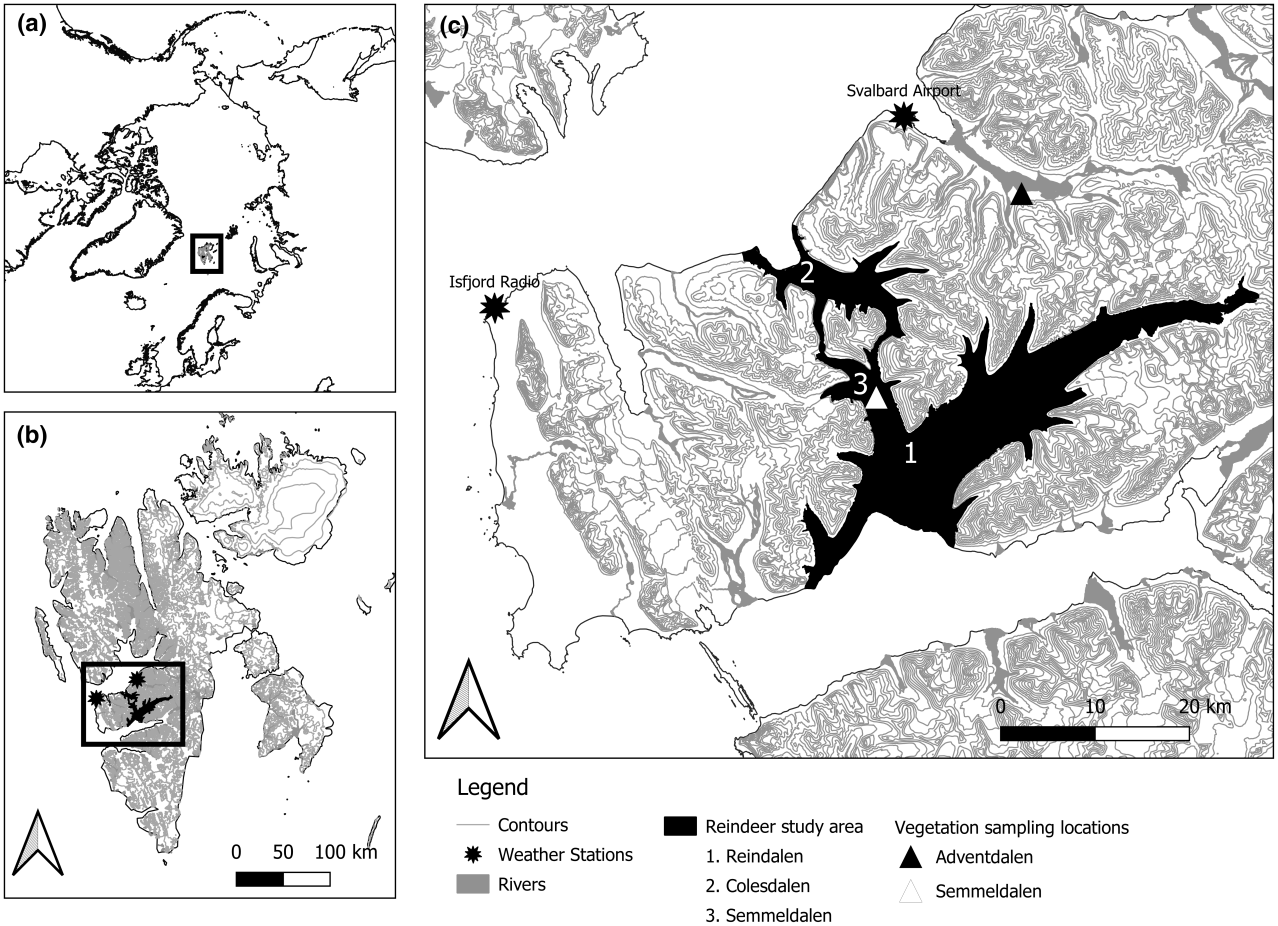
Location of the study area. (a) A map showing the Arctic with Svalbard delineated with a black polygon and (b) a map of Svalbard with Nordenskiöldland delineated with a black polygon. Map lines delineate study areas and do not necessarily depict accepted national boundaries. (c) The study area, the Reindalen valley system, consists of the valleys Reindalen (1), Colesdalen (2) and Semmeldalen (3). Black stars indicate the locations of the Isfjord Radio and Svalbard Airport meteorological stations, and the triangles indicate the vegetation sampling locations in Adventdalen (black) and Semmeldalen (white).

The study was carried out in Nordenskiöld Land, western Svalbard, in the Reindalen valley system (77°–78°N, 15°–17°E), which consists of three main valleys, Colesdalen, Reindalen, and Semmeldalen, as well as smaller side valleys (Figure 1). The vegetation is continuous and dominated by low‐statured graminoids, dwarf shrubs, and forbs over moss carpets (van der Wal & Stien, 2014). The climate on western Svalbard is relatively mild for the latitude, and the average July temperature on the coast is 4.8°C (Isfjord Radio, 78°4′N, 13°37′E) and in the fjord zone, Longyearbyen, 5.9°C (Svalbard Airport, 78°15′N, 15°28′E; Johansen et al., 2012). Average February temperatures are −12.4°C (Isfjord Radio) and −16.2°C (Svalbard Airport; Johansen et al., 2012). The average annual temperatures between 1988 and 2017 increased by 1.7°C compared to the reference period 1971–2000 (Svalbard Airport; Hanssen‐Bauer et al., 2019).

Annual precipitation varies across the archipelago and is 337 mm on the W coast and decreases to 190 mm in the fjord areas (Førland et al., 2011; Johansen et al., 2012; Norwegian Meteorological Institute, 2021a). Between 1971–2017, the average precipitation increased by 4.1% (~4.1 mm) per decade (Svalbard Airport; Hanssen‐Bauer et al., 2019). In winters, the frequency of precipitation events during above‐zero temperature periods, ROS events, has increased (Figure S11; Peeters et al., 2019) and simultaneously, the duration of snow‐covered days has decreased from an average of 253 days (1976–1997) to 221 (2006–2020; Norwegian Meteorological Institute, 2021b).

Svalbard reindeer are found across the non‐glaciated parts of the archipelago (Pacyna et al., 2018; Pedersen, 2017), the highest population densities are in Nordenskiöld Land, Edgeøya, and Barentsøya, but are highly variable between years (Norwegian Institute for Nature Research [NINA], 2021). The Reindalen population increased over the study period (1995–2012) from around 950 female reindeer in the late 1990s to about 1300 female reindeer in 2011–2012 (Figure S10; Albon et al., 2017).

### 2.2 Svalbard reindeer sampling

Reindeer, 3‐ to 15‐year‐old females, were caught annually in late winter (late April to early May) using a net between two snowmobiles and restrained without immobilization drugs as part of a long‐term capture‐mark‐recapture study (Milner et al., 2003; Omsjoe et al., 2009; Stien et al., 2002). During capture, ear tags and neck marker straps on each reindeer were used to record the individual's identity, then live body mass was measured and pregnancy was diagnosed (Ropstad et al., 1999). Blood was collected from the jugular vein into plain vacutainers and centrifuged (2000 RCF, 10 min) within 12 h to retrieve serum. Serum samples were obtained from 1995 to 2012, excluding the years 2003 and 2010, resulting in a total of 16 sampling campaigns. Altogether 232 samples were collected from 182 individual reindeer, thus, some individuals were sampled only once and others up to three times during our study period. The number of samples per campaign ranged from 10–21, with the average value being 15. Serum samples were stored frozen at −80°C until freeze‐drying (48 h, −45°C, 8 mbar, Modulyo 4 K, Edwards).

### 2.3 Forage sources for diet reconstruction

The proportion of the δ^13^C and δ^15^N isotopes in reindeer blood serum is expected to reflect the diet of the reindeer in the weeks preceding sampling, that is, late winter (Dalerum & Angerbjörn, 2005; Halley et al., 2010; Tieszen et al., 1983). For reconstructing the diets of Svalbard reindeer, foliar δ^13^C and δ^15^N values of plausible (terrestrial) forage plants, including graminoids, mosses, dwarf shrubs (*S. polaris*; *D. octopetala*), and forbs were obtained from previous studies (Hansen, Lorentzen, et al., 2019; Zhao et al., 2019). Briefly, plant samples were collected in Semmeldalen (77°90′N, 15°20′E) during July 2009 and 2013 from *Luzula* heath and graminoid vegetation communities (Zhao et al., 2019), and from reindeer feeding craters in Adventdalen (78°10′N, 16°02′E) in March 2013 (Hansen, Lorentzen, et al., 2019). Foliar δ^13^C and δ^15^N values may vary over time due to differences in environmental conditions (Dawson et al., 2002). To control for this, vegetation was re‐sampled in Adventdalen in February 2019 from five randomly selected 4 m^2^ plots as no feeding craters were observed due to extreme ground icing. The plant samples collected were graminoids (*n*  = 2), mosses (*n* = 4), *S. polaris* (*n* = 2), and *D. octopetala* (*n* = 5). Forbs (*n* = 4) were collected as they were present in the plots but not present in or collected from the craters sampled in 2013. All plant samples were stored frozen (−20°C) until drying (60°C, 48 h).

### 2.4 Stable isotope analysis

The dried serum and plant samples were homogenized in a TissueLyser II (Qiagen GmbH) at 25 Hz with 5‐mm stainless steel beads (Qiagen GmbH) that were rinsed with 100% ethanol and dried between samples. Powdered serum (0.8–1.2 mg) and forage (3.5–4.5 mg) samples were weighed into 3.5 × 5 mm tin capsules (Elemental Microanalysis Ltd, UK). The serum samples were analysed at the Environment and Natural Resources Institute Stable Isotope Laboratory at the University of Alaska Anchorage (http://www.uaa.alaska.edu/enri/labs/sils) and the 2019 forage samples at the EcoCore Analytical Facility at the Colorado State University (https://ecocore.nrel.colostate.edu/ecocore‐stable‐isotope‐analysis.html). δ^13^C and δ^15^N values were calibrated against internal standards (BWBII keratin, peach leaves, moose [*Alces alces*], and three‐spined stickleback [*Gasterosteus aculeatus*]). All values were referenced to the international standards of Vienna Pee Dee Belemnite for δ^13^C and air for δ^15^N. Analytical error for the samples was ±0.1‰ for both C and N. Anthropogenic inputs of C from fossil fuel burning has resulted in measurable decreases in atmospheric δ^13^C known as the “Suess effect” (Ben‐David & Flaherty, 2012) and some authors use a time‐dependent correction of −0.022‰ per year based on ice core records to account for this change (Hopkins & Ferguson, 2012). However, the discrimination of δ^13^C during photosynthesis in C_3_ plants has been found to increase in response to increasing concentrations of atmospheric CO_2_ (*p*CO_2_) and decreasing atmospheric δ^13^C_CO2_ (Schubert & Jahren, 2012). Thus, we used the equation of Schubert and Jahren (2012) and annual *p*CO_2_ (Tans & Keeling, 2022) to correct all serum and forage samples δ^13^C values to expected 2012 levels. The full data set compiled for the analyses that follow are available from the Dryad Digital Repository (Hiltunen et al., 2022).

### 2.5 Data analyses

All analyses were done in R version 4.05. (R Core Team, 2021) in R Studio (version 1.4.1106).

### 2.6 Svalbard reindeer diet reconstruction

The proportions of the different forage sources in the diet over time were estimated using Bayesian stable isotope mixing models in *simmr* (R‐package *simmr* version 0.4.5; Parnell, 2019). The performance of these mixing models is sensitive to the selection of priors and when available, informative priors are recommended (Phillips et al., 2014). Informative priors were available from forage species found in Svalbard Reindeer rumen contents collected in late winter (April/May) during 2000–2002 by Bjørkvoll et al. (2009). The mean percentage of forage species were as follows: graminoids (32%), mosses (26%) and *S. polaris* (24%), forbs (4%) and *D. octopetala* (4%; Table S1).

Tissue discrimination factors (TDFs) or the change in isotope ratios between source and consumer are often the weakest link in the reconstruction of diets (Bond & Diamond, 2011). In this study, TDFs were estimated using *SIDER* (version 1.0.0.0), an R package that incorporates reported TDFs, phylogenetic relatedness, foraging ecology, and tissue type into the Bayesian imputation of the most likely TDF values (Healy et al., 2017). The imputed TDFs were ∆^13^C = 4.51 ± 1.94‰ and ∆^15^N = 3.36 ± 1.24‰.

Bayesian stable isotope mixing models are also sensitive to the forage source data used. As forage samples were not available for the same years and locations as the reindeer serum samples, the available forage data were evaluated and grouped according to best practice principles (Phillips et al., 2014). That is all used plant sources are included, and they match the consumer's samples spatially, seasonally, and temporally as closely as possible (Table S2). Four combinations of the source data (Table S3) were tested in the stable isotope mixing models. The *simmr* models were fitted to the reindeer serum data from each year separately using a burn‐in period of 5000 MCMC iterations out of a total of 130,000 iterations of four chains that were thinned to every 50th iteration before analysis (Parnell, 2019; Parnell et al., 2013). Convergence was evaluated using the Brooks‐Gelman‐Rubin diagnostics (Fernández‐i‐Marín, 2016). Posterior distributions, posterior predictive values, credible intervals (CIs), correlation matrices and the deviance information criterion (DIC) were all used to check the performance of all the models. The models run with the source data from all three studies combined (Table 1) were found to be the best performing in terms of having the lowest DICs and correlation matrices within acceptable ranges (Figures S1–S6; Tables S4–S7).

**TABLE 1:** The δ^13^C and δ^15^N values (‰) of the forage plant species (*Dryas octopetala*, *Salix polaris*) and plant groups (forbs, graminoids, mosses) used in the stable isotope mixing models to reconstruct the late winter diets of Svalbard reindeer. The values present mean ± SD and the data were obtained from Hansen, Lorentzen, et al. (2019), Zhao et al. (2019) and samples collected during the current study in the late winter of 2019

Forage group	*n*	δ^13^C	δ^15^N
*D. octopetala*	17	−30.79 ± 0.84	−5.83 ± 1.60
Forbs	13	−29.81 ± 1.30	−4.38 ± 1.82
Graminoids	42	−29.14 ± 1.54	2.07 ± 3.16
Mosses	13	−27.97 ± 1.15	−3.37 ± 0.90
*S. polaris*	16	−29.57 ± 0.93	−4.18 ± 1.14

### 2.7 Svalbard reindeer isotopic niche space reconstruction

The isotopic niche spaces of the reindeer were reconstructed for all years using *SIBER* (Stable Isotope Bayesian Ellipses in R package; version 2.1.5; Jackson et al., 2011). Standard ellipse areas after small sample size correction (SEA_C_) have a 95% probability of containing sampled parameters and were plotted with δ^13^C and δ^15^N values in bivariate δ‐space. We used Bayesian standard ellipse area (SEA_B_) estimates from 10^4^ replicates to test for differences between the years through a comparison of the proportion of posterior ellipses (PP) that differed between groups. Differences in SEA_B_ were considered significant if PP ≥0.95. In addition, the Bayesian CIs derived from the SEA_B_ were compared to determine if there were interannual differences in niche breadths (Cheeseman et al., 2021). Overlaps between the estimated 95% prediction ellipses of all the years were determined. Due to the large number of ellipses and comparisons, we then focused on two pairs of consecutive years at the beginning (1995 [*n* = 15]; 1996 [*n* = 10]) and end (2011 [*n* = 17]; 2012 [*n* = 21]) of the study where the first year had no or little ROS and the second year had extreme ROS (>60 mm).

### 2.8 Drivers of the temporal trends in δ^13^C and δ^15^N values of Svalbard reindeer

As the isotopic signatures varied over the years, we, firstly, performed a post hoc test to inspect for significant differences between the years (Tukey's HSD; *α* = .05). Then, we ran a selection of linear mixed‐effects models (LMMs) to explore the underlying drivers of the variation in the response variables δ^13^C and δ^15^N (*lme*; R‐package *lme4* version 1.1‐27.1; Bates et al., 2015). This modelling approach is recommended as it can account for repeated observations from the same individual as well as spatial and temporal autocorrelation (Zuur et al., 2009). To account for the repeated measurements of individuals between years as well as the environmental variables only differing between years; reindeer identity (ID) and year were fitted as random factors using restricted maximum likelihood. The intrinsic factors of nutritional status and pregnancy that are known to affect the response variables were incorporated into the null models as fixed effect predictor variables. As a proxy for nutritional status, we used body mass (Figure S9) in both the δ^13^C and δ^15^N models while pregnancy status (presence/absence) was used only in the δ^15^N models.

The extrinsic fixed‐effect predictor variables used were: (1) Population density in the Reindalen valley system the previous summer to account for density‐dependent competitive effects (Figure S10; Albon et al., 2017). (2) ROS (mm), measured as the sum of precipitation when the average daily temperature was ≥1°C during the winter (1 November to the 31 March), as a proxy for ground ice (Figure S11; Peeters et al., 2019). (3) July average air temperatures at Svalbard Airport, obtained from the Norwegian Meteorological Institute (2021c), as a proxy for summer plant biomass production (Figure S12; van der Wal & Stien, 2014). Population density and ROS were ln‐transformed before being fitted as predictor variables in the model for δ^13^C. Six biologically relevant candidate models for variation in δ^13^C and δ^15^N were developed based on the abovementioned predictor variables (Table S12). Models were compared using the conditional Akaike information criterion (cAIC) as implemented for the ‘*lme4*’ package in R in the package “*cAIC4*” (R package *cAIC4* version 1.0; Vaida & Blanchard, 2005). Models with a ΔcAIC ≤2 were considered equally relevant. The proportion of variance explained (*R* ^2^) by the fixed factors alone (marginal *R* ^2^, $R_{\mathrm{LMM}\left(m\right)}^{2}$) and by both the fixed and random factors (conditional *R* ^2^; $R_{\mathrm{LMM}\left(c\right)}^{2}$) were estimated using the *rsquared* function in the package “*piecewiseSEM*” (R package *piecewiseSEM* version 2.1.2, R; Nakagawa et al., 2017). The fit of the selected models was assessed graphically by examining the distribution of the residuals against the fitted values and quantiles (R package *redres* version 0.0.0.9; Goode, 2019).

## 3 RESULTS

**FIGURE 2:**
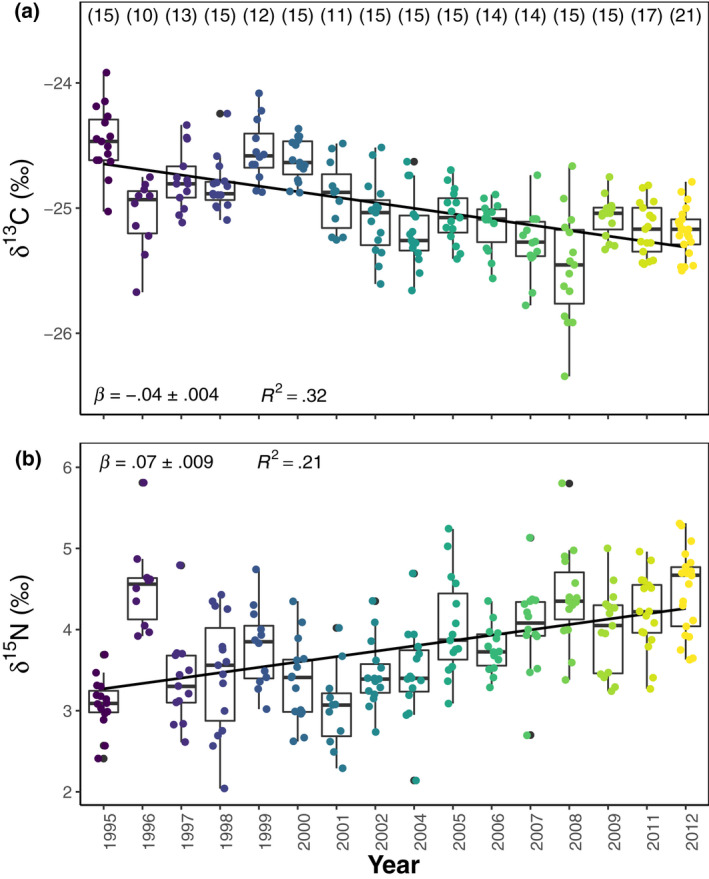
The stable isotope values (a) δ^13^C and (b) δ^15^N in the serum of female Svalbard reindeer (*n* = 232) collected in the Reindalen valley system, Svalbard, between 1995 and 2012. Boxes and whiskers show median, 25%–75% range, interquartile and non‐outlier range with the filled circles indicating observations from individual reindeer. The number of observations for each sampling year is indicated within brackets and panels (a, b) include fitted linear regressions. Note that the years 2003 and 2010 had no samples.

Isotopic ratios from serum exhibited interannual variability with δ^13^C predominantly showing a negative temporal trend (estimates slope, *β* = −.05‰, SE = .004, *p* < .001, Figure 2a) and δ^15^N showing a positive temporal trend (estimates slope, *β* = .07‰, SE = .009, *p* < .001, Figure 2b) over the 17‐year study period. The pattern of variation resulted in a strong correlation between annual average values of δ^13^C and δ^15^N (*r* = −.65, *p* = .007).

### 3.1 Reconstructed diets of Svalbard reindeer based on mixing models

**FIGURE 3:**
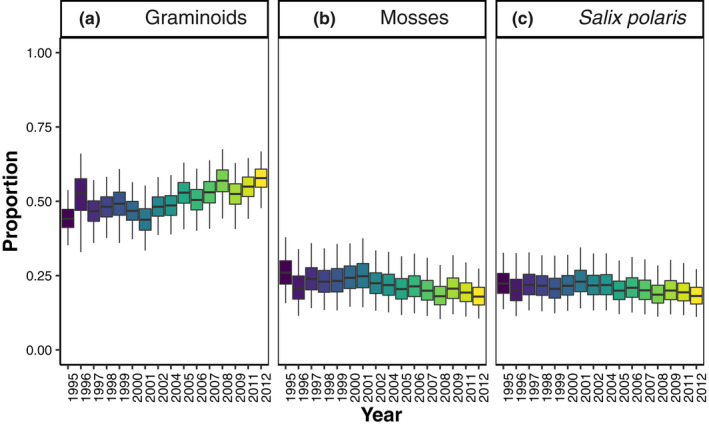
Contributions of (a) graminoids, (b) mosses, and (c) *Salix polaris* to the diets of Svalbard Reindeer between 1995 and 2012. Note that the years 2003 and 2010 had no samples. The diets were reconstructed from the δ^13^C, and δ^15^N values of female Svalbard reindeer (*n* = 232) serum collected in the Reindalen valley system, Svalbard. The dietary proportions are presented in box and whisker plots where boxes show medians and the 25%–75% CI range, and whiskers show 2.5%–97.5% CI range. The diet reconstruction was modelled using the R‐package *simmr* (version 0.4.5, Parnell, 2019). For more details, see Supporting Information 1 and Table S7.

Dietary compositions reconstructed by the selected stable isotope mixing model showed interannual variation (Figure 3; Table S7). Graminoids were dominant in the late winter diets of the reindeer between 1995 and 2012. The proportion of graminoids showed a positive temporal trend, increasing from 44% (95% CI: 35–54) in 1995 to 58% (95% CI: 48–67) in 2012 (Figure 3a; Table S7). The consumption of mosses showed a negative temporal trend, decreasing from 26% (95% CI: 16–38) in 1995 to 18% (95% CI: 11–24) in 2012 (Figure 3b; Table S7). The proportions of *S. polaris* (Figure 3c; Table S7) remained relatively stable between 1995 (22%, 95% CI: 14–33) and 2012 (18%, 95% CI: 11–27). The contribution of forbs and *D. octopetala* were low and did not change over the study period (Table S7).

### 3.2 Svalbard reindeer isotopic niche widths

**FIGURE 4:**
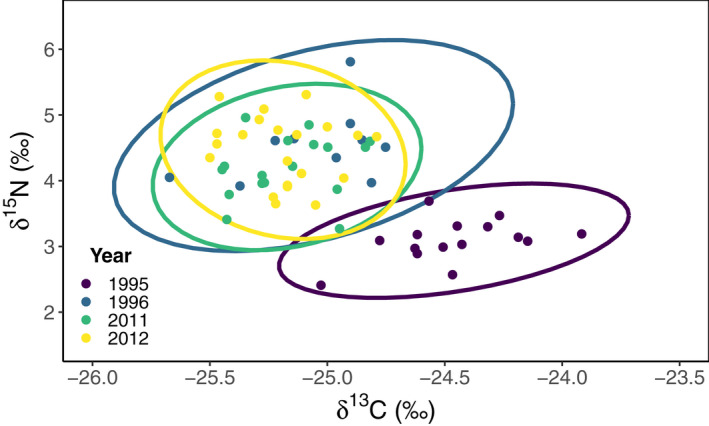
Isotopic niche spaces of female Svalbard reindeer in the Reindalen valley system, Svalbard during consecutive pairs of years at the beginning (1995 and 1996) and the end (2011 and 2012) of the study. In both pairs of years, the first year (i.e., 1995 and 2011) had no or little rain‐on‐snow (ROS) and the second year (i.e., 1996 and 2012) had extreme ROS (>60 mm). The filled circles indicate serum δ^13^C and δ^15^N values of individual reindeer for the different years whereas the ellipses are the SIBER standard ellipse areas (SEA_C_) with 95% confidence intervals.

The isotopic niches reconstructed from δ^13^C and δ^15^N values of Svalbard reindeer showed isotopic drift over time with high interannual variation in overlaps (Figure S7; Table S10). In addition, all year specific ellipses (SEA_B_) differed significantly in size from at least one other year (Figure S8; Table S9). The smallest isotopic niche width was for 2006 (SEA_B_ = 0.19; 95% CI: 0.08–0.72; *n* = 14) while the largest was for 2008 (SEA_B_ = 0.80; 95% CI: 0.32–3.09; *n* = 15). The average proportion overlap in the period between 1995 and 2000 was 36%, between 2001 and 2006 the overlap increased to 42% and then further increased to 53% between 2007 and 2012. Extreme ROS events of greater than 60 mm of precipitation were observed in 1996 and 2012, and the overlap between these two years was 57% (Figure 4; Table S10). In contrast, the overlap between these extreme ROS years with the years prior (1995 and 2011), when no ROS was observed, was 0% between 1995 and 1996, and 71% between 2011 and 2012.

### 3.3 Drivers of the temporal trends in δ^13^C and δ^15^N values of Svalbard reindeer

Among the six candidate models describing the relationship between intrinsic and extrinsic factors and δ^13^C, we identified three models with ΔcAIC ≤2 (Table S11). Of these models, the full model that included ROS, July average temperature, population density and body mass was selected as a parsimonious model (∆cAIC = 1.75; Table S11). The total variance explained by the fixed effects ($R_{\mathrm{LMM}\left(m\right)}^{2}$) in the selected δ^13^C model was only 27%, while 64% of the variance was explained by both the fixed and random effects ($R_{\mathrm{LMM}\left(c\right)}^{2}$; Table S11). In the selected model, δ^13^C decreased as ROS, July average temperature, population density and body mass increased, but the effect of July average temperature was not significantly different from zero (Table 2). The fixed effect predictor variables explained some of the temporal trend in average δ^13^C, but not all. The correlation between year and average δ^13^C was *r* = −.75 in the raw data and was reduced to *r* = −.41 when δ^13^C levels were estimated by the annual average residual values, corrected for estimated fixed effects.

**TABLE 2:** Parameter estimates (*β* ± SE) from the selected linear mixed‐effects models assessing the effects of extrinsic—rain‐on‐snow (ROS), July average temperature and population density—and intrinsic—body mass and pregnancy (only for δ^15^N)—predictors on δ^13^C and δ^15^N values of female Svalbard reindeer. Female identity (ID) and year were included as random intercept effects and natural logarithmic (ln) transformation was used for ROS and population density in the δ^13^C model

Model	Random effects	Variance	SD	Fixed effects	Estimate	SE	*t* Value
δ^13^C	ID	0.0080	0.0892	Intercept	−18.9653	2.8355	−6.689
	Year	0.0474	0.2177	ln(ROS)	−0.0755	0.0569	−1.328
	Residual	0.0552	0.2350	July average temperature	−0.0200	0.0766	−0.261
				ln(Population density)	−0.7677	0.4427	−1.734
				Body mass	−0.0072	0.0028	−2.530
δ^15^N	ID	0.1161	0.3408	Intercept	0.8846	0.7452	1.187
	Year	0.1073	0.3276	ROS	0.0150	0.0050	2.981
	Residual	0.1145	0.3384	July average temperature	0.2039	0.1185	1.721
				Population density	0.0004	0.0007	0.628
				Pregnancy	−0.5031	0.0815	−6.176
				Body mass	0.0222	0.0066	3.362

*Note*: Predictors with non‐significant parameter estimates are highlighted in grey.

Four of the six candidate models identified to explain the temporal variance in δ^15^N in response to the intrinsic and extrinsic factors were relevant as their ΔcAIC ≤2 (Table S11). As with the δ^13^C, the full model that included ROS, July average temperature, population density, body mass and pregnancy was among these parsimonious models. The total variance in δ^15^N explained by the full model ($R_{\mathrm{LMM}\left(c\right)}^{2}$) was 78% and by the fixed effects alone ($R_{\mathrm{LMM}\left(m\right)}^{2}$) alone was 34% (Table S11). Increases in ROS, July average temperature, population density and body mass were all associated with increased δ^15^N while pregnancy was associated with reduced δ^15^N (Table 2). The parameter estimate for the effect of population density was not significantly different from zero (Table 2). As for δ^13^C, the fixed‐effect predictor variables explained some of the temporal trend in average δ^15^N, but not all. The correlation between year and average δ^15^N was *r* = .58 in the raw data and was reduced to *r* = .32 when δ^15^N levels were estimated by the annual average residual values, corrected for estimated fixed effects. Of all the predictors in the selected models for both δ^13^C and δ^15^N, only population density and July average temperature showed a marked trend over the study period and are therefore driving some of the temporal trends in δ^13^C and δ^15^N.

## 4 DISCUSSION

In this study, we sampled the serum of Svalbard reindeer between 1995 and 2012 to identify how the winter diets of a High Arctic herbivore have changed in response to long‐term climate trends and interannual variation, mostly depicted by the “normalization” of ROS events, increased summer temperatures and increased population density. Preceding work has reported short‐term variations in the summer and winter diets of Svalbard reindeer (Bjørkvoll et al., 2009; Hansen, Lorentzen, et al., 2019; Pacyna et al., 2018; Zhao et al., 2019). We demonstrate changes in the form of long‐term trends in the winter diets.

### 4.1 Temporal increase in graminoid consumption by Svalbard reindeer

We expected reindeer winter diets to follow OFT and thus the changes in the abundance and availability of forage species. Consequently, dietary shifts would be expected to parallel the observed increases in greening (Vickers et al., 2016), biomass production, and abundance in graminoids (Hansen et al., 2010b; van der Wal & Brooker, 2004) on Svalbard and shrubification across the Arctic (Myers‐Smith et al., 2011, 2020). Accordingly, we found strong support for increased proportions of graminoids (44% → 58%) and decreased proportions of mosses (26% → 18%) in the reindeer late winter diets over time, but contrary to our expectations, the proportion of *S. polaris* did not increase (Figure 3). We also found that the reconstructed isotopic niches of Svalbard reindeer shifted, expanded, and contracted over the years with different ROS conditions in a manner suggesting dietary adaptation to more frequently occurring ROS and an increasing abundance of graminoids. Furthermore, the data suggested that the isotopic niche metrics differed in response to ROS in the early years of our study period in comparison to the later years. Lastly, we found that population density together with climate, that is, summer air temperature and ROS, were important drivers of serum isotopic variability highlighting the important role of both density‐dependent effects and the changing climate for the physiology and realized trophic niche of these herbivores. Similar to our findings climatic patterns, such as the broad scale Arctic Oscillation, have been found to influence caribou and reindeer body condition and subsequently population dynamics through effects on the quality of the summer‐ranges Svalbard (Aanes et al., 2002), in Alaska (Joly et al., 2011) and in northern Canada (Mallory et al., 2018).

Svalbard reindeer are not the only *Rangifer* spp. whose winter diets have shifted toward more graminoids. A similar trend was observed as caribou in the Western Arctic Herd, Alaska consumed more graminoids and fewer lichens when the abundance of lichens in the foraging habitat declined (Joly et al., 2007). However, these findings may not be generalizable across the Arctic, as there is variation in how well the different *Rangifer* subspecies can adapt their diets to changing foraging ranges and forage availability (Mallory & Boyce, 2018). Graminoids are grazing tolerant and adapted to defoliation with basal meristems located close to the soil surface and indeterminate growth allowing continued biomass production after leaf removal by grazers (Barthelemy et al., 2015, 2019; Welker & Briske, 1992). They are also relatively tolerant to trampling while mosses and dwarf shrubs are more sensitive (Egelkraut et al., 2020). Consequently, graminoids benefit both from increased tundra soil temperature and increased soil nutrient availability generated by higher summer temperatures, a reduction in the insulating shrub and moss layer, and more nutrients released from reindeer faeces and urine (Aerts et al., 2006; Barthelemy et al., 2019; Egelkraut et al., 2020; Marino et al., 2014; Milner et al., 2018; Parsons et al., 1995; Sjögersten et al., 2012; van der Wal et al., 2004; van der Wal & Brooker, 2004). Via these mechanisms, reindeer grazing may induce vegetation state transitions from moss dominated tundra to graminoid dominated tundra thereby increasing the ecosystem productivity and forage quality for reindeer (van der Wal, 2006; Ylänne et al., 2018). The increased abundance of graminoids due to increased grazing pressure and climate change (van der Wal & Brooker, 2004) may facilitate Svalbard reindeer survival, especially during winters with ROS. Graminoids are not damaged by ice encasement or fluctuating winter temperatures (Bjerke et al., 2017, 2018) and due to their erect growth form (Parsons et al., 1995), they may protrude above the ice layers thus remaining accessible to herbivores. Indeed, on Svalbard, increased graminoid and decreased moss abundance correlates positively with Svalbard reindeer density and increased grazing pressure (Hansen et al., 2010b; van der Wal & Brooker, 2004).

Contrary to our prediction, the proportion of *S. polaris* was stable with slight interannual variations in the reindeer winter diets between 1995 and 2012 (Figure 3). These results were surprising, as *S. polaris* is an abundant species in exposed habitats (ridges and heaths; Le Moullec et al., 2019; van der Wal & Stien, 2014) and even when encased in ice or exposed to erratic winter temperatures remains undamaged (Le Moullec et al., 2020). Furthermore, Bjørkvoll et al. (2009) observed an increase in *S. polaris* (9%) in the late winter rumen contents between 2000 and 2002. Interestingly, the estimated mean proportions of *S. polaris* in 2001 were the same (23%) in both our and Bjørkvoll et al.'s (2009) studies even though the methods differ substantially. The rumen contents and faecal pellets analysed by Bjørkvoll et al. (2009) tend to inflate the contributions of poorly digestible mosses and woody plants (*S. polaris*) at the expense of highly digestible graminoids (Bjørkvoll et al., 2009; Gaare et al., 1977; Staaland, 1984). SIA only detects the contributions of δ^13^C and δ^15^N from digested and assimilated forage, and may, therefore, underestimate the use of the poorly digestible plants (Ben‐David et al., 2001). Nonetheless, there appears to be no change in the contribution of *S. polaris* to Svalbard reindeer diets. Thus it may be that the prostrate woody stems and the senescent leaves on the ground are ice‐encased and inaccessible while in the ice‐free exposed habitats the reindeer are actively selecting against *S. polaris* in favour of higher‐quality graminoids. These patterns in Svalbard reindeer diets follow the premises of OFT under all scenarios and point toward increasing proportions of graminoids in their winter diets.

### 4.2 Isotopic niche shifts by Svalbard reindeer

The two‐dimensional isotopic niches reconstructed from the Svalbard reindeer serum δ^13^C and δ^15^N values can be used as a proxy for the trophic niche (Bearhop et al., 2004). We found that these winter isotopic niches had drifted over time (1995–2012), with high interannual variation in position, breadths (SEA_B_) and overlaps. This suggests that the winter foraging niche of Svalbard reindeer has been changing for decades and not only in response to an increased frequency in ROS events (Beumer et al., 2017). Our comparisons of reconstructed isotopic niches from subsequent high and low ROS winters in the early and later phases of our study suggest that the foraging strategy of Svalbard reindeer in low ROS years may have changed over time to become more like the strategy adopted in high ROS years. The temporal trend in the isotopic niche variance is most likely due to the increased consumption of graminoids, especially when taking into consideration the observed temporal decline in global foliar δ^15^N (Mason et al., 2022). However, the interannual isotopic niche variance may be due to the varying availability, selection, and utilization of different combinations and proportions of graminoid species with contrasting isotopic values such as *Alopecurus alpina* (δ^13^C: 28.32 ± 1.3; δ^15^N: 4.95 ± 3.8) or *Luzula confusa* (δ^13^C: −30.65 ± 0.8; δ^15^N: 1.63 ± 3.0; Matthews & Mazumder, 2004; Staaland, 1984; Yeakel et al., 2016; Zhao et al., 2019).

### 4.3 Extrinsic drivers of the shifts in Svalbard reindeer serum isotope values and diets

Preceding work has reported contrasting changes in caribou δ^13^C and δ^15^N values during winters with body mass declines (Barboza et al., 2020; Gustine et al., 2014; Parker et al., 2005) and fat and protein reserves are mobilized in response to declining energy and N intake (Mänttäri et al., 2013; Reimers, 1984). In the current study, a low body mass was on average associated with enriched δ^13^C values and depleted δ^15^N values, although the effect was small (Table 2). In contrast, in 1996 when an extreme ROS event resulted in severe starvation and relatively high mortalities (Milner et al., 2003) we observed low body masses, serum δ^13^C depletion and δ^15^N enrichment (Figure S9; Figure 2). Still, our results support the findings of previous studies, in that they suggest that intrinsic processes, such as pregnancy, and nutritional status (mobilization of body stores) have small contrasting effects on serum δ^13^C and δ^15^N values. Dietary supplies of energy and N are the largest contributors to the stable isotope pool in serum (Ben‐David & Flaherty, 2012; Fry, 2006; Rogers et al., 2015, 2020; Stanek et al., 2017, 2019), consequently, shifts in the serum δ^13^C and δ^15^N values are most likely due to increased utilization of δ^15^N enriched and δ^13^C depleted graminoids in late winter. Ultimately, we propose that the main extrinsic environmental drivers of Svalbard reindeer serum δ^13^C and δ^15^N values—population density, summer temperature, and ROS—have acted collectively to increase the utilization of graminoids in later winter.

## 5 CONCLUSIONS AND FUTURE IMPLICATIONS

This long‐term study highlights that the foraging behaviour of Svalbard reindeer in late winter is changing. Previous studies have shown that the increased forage abundance in summer and the lengthening of the growing season is likely to counteract the negative effects of more frequent harsh winters, which traditionally have caused forage shortages (Mallory & Boyce, 2018; Mallory et al., 2020). Our study shows that changes in the winter foraging behaviour suggest that higher‐quality food is available. Together these changes in the forage quantity, quality, and availability during all seasons are all likely to have positive consequences for the diet and population growth of Svalbard reindeer and have contributed to the doubling of the Reindalen population from 1996 (*n* ~733) to 2014 (*n* ~1758; Albon et al., 2017) as well as the continued increase in numbers thereafter (NINA, 2021). The responses of other *Rangifer* populations to the rapidly changing climate in the Arctic are currently, and also expected to, be varied, thus extreme care should be taken when extrapolating the results of the current study to other *Rangifer* subspecies (Mallory & Boyce, 2018). Nonetheless, environmental change seems at present to be in a phase where conditions are improving for one of the northernmost *Rangifer* populations in the world and has enabled their late winter diet to change from low‐quality mosses toward increasingly more available higher‐quality graminoids.

## AUTHOR CONTRIBUTIONS

Jeffery M. Welker and Audun Stien conceived, designed, and initiated the study. Erik Ropstad collected and stored the blood samples from a previous study, and Jeffery M. Welker and Tamara A. Hiltunen collected forage samples. Audun Stien and Tamara A. Hiltunen performed the LMMs. Tamara A. Hiltunen analysed the isotope data, performed the isotopic niche and mixing models, and led the development of the figure presentations. Tamara A. Hiltunen, Maria Väisänen, Audun Stien and Erik Ropstad interpreted the data and led the writing of the manuscript. Jeffery M. Welker and Jouni O. Aspi provided supervision throughout the project and writing phases. All authors contributed critically to the drafts and gave approval for publication.

## FUNDING INFORMATION

The work was supported mainly by grants from U.K. Natural Environment Research Council (GR3/1083), the Norwegian Research Council and the Macaulay Development Trust. Additional financial support has come from the Amundsen Foundation, Centre for Ecology and Hydrology, the Macaulay Institute, NINA, UNIS, and the Norwegian School of Veterinary Science. Funding for JMW was supported by the Distinguished US Arctic Research Chairship‐Norway and the Inaugural UArctic Research Chairship. The analyses were made possible in part by a National Science Foundation Major Research Instrumentation award (0953271) to JMW. Funding for TH and MV was supported by JMW's UArctic Research Chairship.

## CONFLICT OF INTEREST

The authors declare no conflicts of interest.

## PERMITS

Reindeer sample collection met all guidelines of the Research Council of Norway and were collected with the permission of the Governor of Svalbard (Sysselmannen).

## Supporting information


Appendix S1

## Data Availability

The data and R scripts that support the findings of this study are openly available in Dryad Digital Repository at https://doi.org/10.5061/dryad.ghx3ffbs7 (Hiltunen et al., 2022). The R scripts are available in Zenodo at https://doi.org/10.5281/zenodo.7049837.
