# Supplementary material for: Svalbard reindeer winter diets: Long‐term dietary shifts to graminoids in response to a changing climate
Source: Glob Chang Biol. 2022 Sep 17;28(23):7009–22. doi: 10.1111/gcb.16420 (PMC9826046; doi:10.1111/gcb.16420)
Supplement: Supplementary file 1 — Appendix S1 [file GCB-28-7009-s001.pdf]

Supplementary Information: Svalbard reindeer winter diets: long-term dietary shifts to graminoids in response to a changing climate.

Table of Contents

1. Diet proportion modelling using stable isotope mixing models in simmr 8
   1.1 Informative priors for diet proportion modelling using stable isotope mixing models 8
   1.2 Determination of the best forage source data to use for stable isotope mixing models 9
   1.3 Stable isotope mixing model (simmr) outputs 15
      1.3.1 Model diagnostics 16
      1.3.2 Matrix plots 17
      1.3.3 Dietary proportion estimates: credible intervals 22
2. Isotopic niche widths & overlaps modelled in SIBER 23
   2.1 Summary Statistics 24
3. Linear Mixed Effects Models 29
   3.1 Fixed Factors 29
      3.1.1 Body Mass of Samples Svalbard Reindeer through the study period 29
      3.1.2 Svalbard Reindeer Population in Reindalen 30
      3.1.3 Rain-on-Snow 31
      3.1.4 Temperature 32
   3.2 Model Selection 33
4. References 34

Table of Supplementary Figures

Figure S1: Biplot of Svalbard Reindeer serum $\delta^{13}C$ and $\delta^{15}N$ and their winter food sources that have been corrected for trophic discrimination for the four separate models that were created to evaluate the best source means to utilize. The different models are shown in panels: (a) model 1; (b) model 2; (c) model 3 and (d) model 4. All forage and serum $\delta^{13}C$ values were corrected to 2012 using the method outlined in Schubert & Jahren (2012). 13

Figure S2: Biplot of Svalbard Reindeer serum $\delta^{13}C$ and $\delta^{15}N$ and their winter food sources (4=W_9_13_19) that have been corrected for trophic discrimination. All forage and serum $\delta^{13}C$ values were corrected to 2012 using the method outlined in Schubert & Jahren (2012). 15

Figure S3: Matrix plot of food sources for the Svalbard Reindeer during the late winter foraging periods of 1995 (a), 1996 (b), 1997 (c) and 1998 (d). The diagonal cells show the posterior probability distributions for each of the food sources (Dryas octopetala, Forbs, Graminoids, Mosses and Salix polaris). The cells below the diagonal show the correlations between contributions for pairs of food sources. The cells above the diagonal show contours of the joint posterior probability distribution for contributions for pairs of food sources........ 18

Figure S4: Matrix plot of food sources for the Svalbard Reindeer during the late winter foraging periods of 1999 (a), 2000 (b), 2001 (c) and 2002 (d). The diagonal cells show the posterior probability distributions for each of the food sources (Dryas octopetala, Forbs, Graminoids, Mosses and Salix polaris). The cells below the diagonal show the correlations between contributions for pairs of food sources. The cells above the diagonal show contours of the joint posterior probability distribution for contributions for pairs of food sources........ 19

Figure S5: Matrix plot of food sources for the Svalbard Reindeer during the late winter foraging periods of 2004 (a), 2005 (b), 2006 (c) and 2007 (d). The diagonal cells show the posterior probability distributions for each of the food sources (Dryas octopetala, Forbs, Graminoids, Mosses and Salix polaris). The cells below the diagonal show the correlations between contributions for pairs of food sources. The cells above the diagonal show contours of the joint posterior probability distribution for contributions for pairs of food sources........ 20

Figure S6: Matrix plot of food sources for the Svalbard Reindeer during the late winter foraging periods of 2008 (a), 2009 (b), 2011 (c) and 2012 (d). The diagonal cells show the posterior probability distributions for each of the food sources (Dryas octopetala, Forbs, Graminoids, Mosses and Salix polaris). The cells below the diagonal show the correlations between contributions for pairs of food sources. The cells above the diagonal show contours of the joint posterior probability distribution for contributions for pairs of food sources..21

Figure S7: Bivariate plots of serum $\delta^{13}C$ and $\delta^{15}N$ showing the SIBER standard ellipse areas surrounding the reconstructed isotopic niche spaces and 95% CIs of female Svalbard reindeer (n=232) from the Reindalen valley system, Nordenskiöldland, Spitsbergen between 1995 and 2012 (excluding 2003 and 2010). The SEAs for the individual years were corrected for small sample sizes (SEA_C).............................23

Figure S8: Boxplot of the sizes of the Bayesian standard ellipse areas (SEA_B) modelled SIBER using the serum $\delta^{13}C$ and $\delta^{15}N$ values. Black dots represent the mode, red crosses represent the mean while the boxes represent 50%, 75%, and 95% credible intervals. The black boxes highlight the pairs of years at the beginning (1995;1996) and end (2011;2012) of the study where the first year had no or little ROS and the second year had extreme ROS (>60mm). See Parnell et al., (2013) for more information on isotopic niche modelling and the associated metrics. 26

Figure S9: Body mass of female Svalbard reindeer (n=232) captured and weighed in the Reindalen valley system, Nordenskiöldland, Spitsbergen between 1995 and 2012 (excluding 2003 and 2010). Box and whisker plots showing body mass values (median, 25%-75%, interquartile range, nonoutlier range and outliers [black points]). Individual observations are represented by the points. Sample sizes of the reindeer are indicated within brackets. 29

Figure S10: Total yearly Svalbard Reindeer population size in Reindalen from 1994 to 2012 with fitted linear regression (black line; Albon et al., 2017)...30

Figure S11: Total yearly rain-on-snow (ROS) for the winters prior to capture and sampling of Svalbard Reindeer from 1995 to 2012. Data obtained from Peeters et al. (2019). The black boxes highlight the pairs of years at the beginning (1995;1996) and end (2011;2012) of the study where the first year had no or little ROS and the second year had extreme ROS (>60mm). 31

Figure S12: July Average temperatures obtained from Svalbard Airport for the period 1994 to 2012 for the summer prior to capture and sampling of Svalbard Reindeer with fitted linear regression. The dashed green line represents the long term at July average temperature for the period 1961 – 1990 (5.9 °C) and the dashed yellow line represents the July average temperature (6.6 °C) for the study period (1994 -2012). Data obtained from the obtained were from the Norwegian Meteorological Institute (MOSJ, 2021)....................32

Table of Supplementary Tables

Table S1: Informative priors for dietary contribution in late winter derived from Bjørkvoll et al. (2009) and the imputed priors modelled in the stable isotope mixing model (simmr). 8

Table S2: Data available for use in mixing mixing models to reconstruct the diets of the Svalbard Reindeer. The different data sets are matched to try and see what the best combinations are to meet best practice principles of mixing models, ie. mix and source matching at utilisation, temporal and spatial scales. Data was obtained from: Hansen et al., (2019)[1] and Zhao et al., (2019)[2] respectively, and samples collected during the current study in the late winter of 2019. 11

Table S3: Source data used in the different models to determine the most appropriate sources to use in the mixing models to reconstruct the diets of the Svalbard Reindeer. Data was obtained from: Hansen et al., (2019)[1] and Zhao et al., (2019)[2] respectively, and samples collected during the current study in the late winter of 2019. All forage δ^{13}C values were corrected to 2012 using the method outlined in Schubert & Jahren (2012). Significant differences in the means between the isotope values of each functional group in the different models are indicated by the symbols ($^{*¤\$@€}$). 12

Table S4: Deviance information criterion (DIC) of the different models to determine the most appropriate sources to use in the mixing models to reconstruct the diets of the Svalbard Reindeer The lowest DIC values are in the light grey boxes. The ΔDIC is calculated between models 1 and 4 and is the higher DIC value minus the lower DIC value. 14

Table S5: Brooks, Gelman, Rubin (BGR) convergence diagnostics for each source and year modelled in the stable isotope mixing model (simmr). The BGR test was used to check for convergence and to evaluate the fitting performance of the models and the values should all be close to 1. All the years modelled showed convergence and good model fit. 16

Table S6: Proportion of observations (y) that lie outside the posterior predictive (**yrep**) of 50% each year modelled with late winter priors in the stable isotope mixing model (simmr). The highest proportion of observations that lie outside **yrep** is 0.467 in 2008, less than the 0.5 specified in the posterior predictive function. For all other years, the proportion of observations that lie outside **yrep** are in the range 0.133 to 0.400, indicating all the models fit well. 16

Table S7: Summary quantiles from the posterior distributions for each of the five sources (Dryas octopetala, Forbs, Graminoids, Mosses and Salix polaris) and years modelled in the simmr package in R to reconstruct the diets of Svalbard Reindeer from the carbon ($\delta^{13}C$) and nitrogen ($\delta^{15}N$) isotopic values. Serum was collected from female Svalbard reindeer (n=232) in the Reindalen valley system, Nordenskiöldland, Spitsbergen between 1995 and 2012 (excluding 2003 and 2010). 22

Table S8: SIBER summary area statistics of the reconstructed isotopic niches using δ13C and δ15N values of female Svalbard Reindeer serum (n=232) from the Reindalen valley system, Nordenskiöldland, Spitsbergen between 1995 and 2012 (excluding 2003 and 2010). Summary statistics include convex hull total area (TA), standard ellipse area corrected for small sample size (SEA_C) and the Bayesian Standard ellipse area (SEA_B). The bold text highlights the pairs of years at the beginning (1995;1996) and end (2011;2012) of the study where the first year had no or little ROS and the second year had extreme ROS (>60mm). 24

Table S9: Probability that isotopic niche area of female Svalbard Reindeer of years in group A are bigger than the years in group B. Isotopic niches were constructed using $\delta^{13}C$ and $\delta^{15}N$ values of reindeer serum (n=232) from the Reindalen valley system, Nordenskiöldland, Spitsbergen between 1995 and 2012 (excluding 2003 and 2010). Values with a PP ≥ 0.95 (yellow highlight) indicates year A is significantly bigger than B while values with a PP ≤ 0.05 (green highlight) indicates that year A is significantly smaller than B. The bold text highlights the pairs of years at the beginning (1995;1996) and end (2011; 2012) of the study where the first year had no or little ROS and the second year had extreme ROS (>60mm). 27

Table S10: Comparison of isotopic ellipse overlaps of Svalbard Reindeer serum $\delta^{13}C$ and $\delta^{15}N$ between all years. Isotopic niches were constructed using $\delta^{13}C$ and $\delta^{15}N$ values of reindeer serum (n=232) from the Reindalen valley system, Nordenskiöldland, Spitsbergen between 1995 and 2012 (excluding 2003 and 2010). The bold text highlights the pairs of years at the beginning (1995; 1996) and end (2011; 2012) of the study where the first year had no or little ROS and the second year had extreme ROS (>60mm). 28

Table S11: Candidate models assessing the variation in $\delta^{13}C$ and $\delta^{15}N$ values of female Svalbard reindeer (n=232) sampled between 1995 and 2012 (excluding 2003 and 2010). Intrinsic predictors were body mass and pregnancy (only $\delta^{15}N$), while extrinsic predictors were rain-on-snow (ROS), July average temperature, and population density. ROS and population density were log-transformed in the $\delta^{13}C$ analysis only. Female

identity (ID) and year were included as random intercept effects. The models were fitted with the default Restricted Maximum Likelihood (REML) and the number of degrees of freedom (K), the conditional Akaike Information Criterion (cAIC) and the difference in cAIC (ΔcAIC) are presented. In addition, the estimated proportion of variance explained (R^2) by the fixed factors alone (marginal R^2, $R^2_{LMM(m)}$) and by both the fixed and random factors (conditional R^2; $R^2_{LMM(c)}$) are presented. The best models (i.e. a ΔcAIC <2) are in black text while the full (selected) models are highlighted in bold text The correlation between year and average $\delta^{13}C$ and $\delta^{15}N$ values in the raw data ($\delta^{13}C$: r = -.78; $\delta^{15}N$: r = .58) was reduced when the $\delta^{13}C$ and $\delta^{15}N$ levels were measured by the annual residual values, corrected for estimated fixed effects ($\delta^{13}C$: r = -.41; $\delta^{15}N$: r = .32). The fixed effect predictor variables in the selected models do not predict all the variance in the temporal trends in $\delta^{13}C$ and $\delta^{15}N$. 33

1. Diet proportion modelling using stable isotope mixing models in simmr

The data and R scripts that support the findings are openly available in Dryad Digital Repository at https://doi.org/10.5061/dryad.ghx3ffbs7

Data: Reindeer_data_schubert.xlsx

Rcode R_Code-simmr_SvalbardReindeer.R

1.1 Informative priors for diet proportion modelling using stable isotope mixing models

Setting of informative prior distributions improves the posterior probability distribution and the parameter estimates and their uncertainty. The priors for these models were derived from a study conducted by Bjørkvoll *et al.* (2009). The study examined the rumen contents of Svalbard Reindeer culled between 2000 and 2002 in late winter (April/May) from the Colesdalen and Semmeldalen valleys (Table S4). The reindeer isotope data in the current study was from serum collected in April/May, and thus reflects the diets in March/April (Ben-David et al., 2001). As dietary proportions must add up to one, the data was corrected to reflect only the species available/ found during the vegetation sampling: *Dryas octopetala*, forbs, graminoids, mosses and *Salix polaris*. Simmr utilizes the loaded priors to impute the best fit estimates for the proportion means and standard deviations. (Table S4).

Table S1: Informative priors for dietary contribution in late winter derived from Bjørkvoll et al. (2009) and the imputed priors modelled in the stable isotope mixing model (simmr).

Forage source	Bjørkvoll (2019)		simmr elicit	
	Mean	SD	Mean	SD
Dryas octopetala	0.05	0.04	0.046	0.039
**Forbs**	0.04	0.03	0.034	0.027
**Graminoids**	0.36	0.09	0.365	0.106
**Mosses**	0.29	0.09	0.296	0.086
Salix polaris	0.26	0.07	0.258	0.069

1.2 Determination of the best forage source data to use for stable isotope mixing models

This study was conducted retrospectively on previously collected and stored Svalbard reindeer serum samples, and as a result samples of forage plants were not available for every year of the study period or even for the study area. Best practice dictates that for stable isotope mixing models, source data should include all utilized forage items and be temporally (i.e. late winter for every year) and spatially (i.e. from the Reindalen valley) relevant to the mixtures being modelled. The following forage items are considered available in late winter in Reindalen to Svalbard reindeer: graminoids, mosses, *S. polaris*, *D. octopetala* and some forbs. Other groups which are utilised by reindeer species in winter such as lichens are virtually absent from the area and others commonly used in summer such as sphenophytes and some forbs completely disappear in early winter. Data from previous studies from late winter 2013 in Advendalen (Hansen et al., 2019) and summer 2009 and 2013 in Colesdalen, Semmeldalen and Reindalen (Zhao et al., 2019) was sourced (Table S1). In addition, forage plants were sampled plants from Advendalen in late winter 2019 as part of the current study. Various combinations of the data from the three studies will be used in the mixing models so that they meet the forage type, spatial and temporal requirements of best practice for the running of mixing models.

Four separate models were created in a stepwise manner to evaluate the best source means to utilize (Table S2). All the $\delta^{13}C$ values were corrected to 2012 using the method outlined in Schubert & Jahren (2012). Model 1 (W_13_19) met the utilisation and seasonal (temporal) criteria but not the spatial and temporal (year) requirements. The second model (WS_13) captures the utilisation, spatial and seasonal (temporal) requirements but not the temporal (year) conditions. The third model (WS_13_19) combines the two previous models and increases the seasonal influence of winter however still fails to capture the temporal (year) requirement. Model 4 (WS_9_13_19) combines all the data available, thus increasing sample sizes for the forage groups, in addition, it captures the requirements of utilisation, spatial, seasonal (temporal) and one year (2009) in the temporal (year) however 15 years are still absent. There were significant differences between the $\delta^{13}C$ means of the mosses for model 1 and all the other models(2: p=0.03, 3:p=0.04, 4: p=2.642e^{-05}), while the $\delta^{13}C$ means of graminoids and *S. polaris* were only significantly different between model 1 and 4 (p=0.008 and p=8.365e^{-05} respectively). Models 1 and 2 $\delta^{13}C$ means for *D. octopetala* were

significantly different (p=0.048). There was no significant difference between the $\delta^{15}N$ means for any of the models or functional groups.

All models were plotted in a bivariate plot of $\delta^{13}C$ and $\delta^{15}N$. All source (plant) and mix (Svalbard Reindeer serum) $\delta^{13}C$ values were corrected to 2012 for the "Suess effect" and when plotted the values of the mix are corrected for trophic enrichment using the selected TDF. Standard practice in evaluating data and its suitability to be modelled in mixing models requires that once the food sources have been corrected for trophic enrichment (discrimination) all the consumer's data must fall within the mixing polygon derived from all sources (Phillips et al., 2014). The current data set was considered suitable for running in the simmr package (Versions 0.4.2) in R as all the data points fall within the mixing polygon (Figure S1).

Stable isotope mixing models were run with the same run parameters in the Materials and Methods, the same prior information (Supplementary: Section 1.2) and trophic discrimination factors (TDFs). The Brooks, Gelman, Rubin (BGR) test was used to check for convergence and to evaluate the fitting performance of the models, and all 16 years in all four models converged. or the. For simmr models, the Deviance Information Criterion (DIC) evaluates how well the model fits the data. Model 4 had the lowest DICs for 10 of the 16 years modelled (1996, 1998, 1999, 2005 to 2012) while model 1 had the lowest DICs for 6 of the 16 years modelled (1995, 1997, 2000, 2001, 2002 and 2004) (Table S3). Thus, according to DIC, the best models are 1 and 4. Correlations among the sources in the matrix plots can suggest a lot about the model uncertainty and possibly help determine the best fitting models in terms of the sources utilized. Very large negative correlations indicate that the model cannot separate the two sources as they are close in isospace, while large positive correlations indicate there are multiple competing sources that are contributing to the mixture (Phillips et al., 2014). The correlation coefficients for many of the years run with the model 1 sources are too high to be acceptable ie they are above 0.75, while the worst performing year for model 4 was 1996 and the correlation was -0.63 which is lower than 0.75. Thus the sources in model 4 are better than model 1. Based on the model diagnostics it was concluded that the model containing all the available source data (Model 4) was the best. The next best model was the source data from winters only in 2013 and 2019 (Model 1). The $\delta^{13}C$ and $\delta^{15}N$ means of graminoids, mosses and *S. polaris* were found to be significantly different between these two data sets

Table S2: Data available for use in mixing mixing models to reconstruct the diets of the Svalbard Reindeer. The different data sets are matched to try and see what the best combinations are to meet best practice principles of mixing models, ie. mix and source matching at utilisation, temporal and spatial scales. Data was obtained from: Hansen et al., (2019)[1] and Zhao et al., (2019)[2] respectively, and samples collected during the current study in the late winter of 2019.

Mixing model mix and source data matching			Mix	Sources		
			**Current**	**Hansen[1]**	**Zhao[2]**	**Current**
**Utilisation**	**Late winter Forage groups**	*Dryas octopetala*	-	√	√	√
		Forbs	-	X	√	√
		Graminoids	-	√	√	√
		Mosses	-	√	√	√
		Salix polaris	-	√	√	√
**Spatial**	**Valley**	Reindalen	√	X	√	X
		Adventdalen	X	√	X	√
**Temporal**	**Season**	Late winter	√	√	X	√
		Summer	X	X	√	X
	**Years**	1995	√	X	X	X
		1996	√	X	X	X
		1997	√	X	X	X
		1998	√	X	X	X
		1999	√	X	X	X
		2000	√	X	X	X
		2001	√	X	X	X
		2002	√	X	X	X
		2004	√	X	X	X
		2005	√	X	X	X
		2006	√	X	X	X
		2007	√	X	X	X
		2008	√	X	X	X
		2009	√	X	√	X
		2011	√	X	X	X
		2012	√	X	X	X
		2013	X	√	√	X
		2019	X	X	X	√

Table S3: Source data used in the different models to determine the most appropriate sources to use in the mixing models to reconstruct the diets of the Svalbard Reindeer. Data was obtained from: Hansen et al., (2019)[1] and Zhao et al., (2019)[2] respectively, and samples collected during the current study in the late winter of 2019. All forage $\delta^{13}C$ values were corrected to 2012 using the method outlined in Schubert & Jahren (2012). Significant differences in the means between the isotope values of each functional group in the different models are indicated by the symbols ($^{*¤\$@€}$).

Model	Data details	Functional Group	n	Mean $\delta^{13}C$	SD $\delta^{13}C$	Mean $\delta^{15}N$	SD $\delta^{15}N$
**1 W_13_19**	Winter 2013[1] Winter 2019	*Dryas octopetala*	9	-30.53*	0.44	-4.93	0.82
		Forbs	3	-29.59	0.62	-4.80	1.19
		Graminoids	6	-29.25#	0.96	0.77	1.23
		Mosses	8	-28.73$@¤	0.36	-3.05	0.70
		Salix polaris	6	-29.49€	0.27	-4.06	0.51
**2 WS_13**	Winter 2013[1] Summer 2013[2]	*Dryas octopetala*	8	-31.27*	0.80	-6.10	2.05
		Forbs	5	-30.37	1.60	-3.90	1.20
		Graminoids	21	-28.96	1.60	1.44	2.82
		Mosses	9	-27.66$	1.24	-3.42	0.95
		Salix polaris	9	-30.02	0.91	-4.11	1.45
**3 WS_13_19**	Winter 2013[1] Summer 2013[2] Winter 2019	*Dryas octopetala*	13	-30.94	0.76	-5.69	1.75
		Forbs	8	-30.08	1.32	-4.24	1.20
		Graminoids	23	-28.91	1.54	1.26	2.77
		Mosses	13	-27.97@	1.15	-3.37	0.90
		Salix polaris	11	-29.93	0.84	-4.13	1.32
**4 WS_9_13_19**	Summer 2009[2] Winter 2013[1] Summer 2013[2] Winter 2019	*Dryas octopetala*	17	-30.79	0.84	-5.83	1.60
		Forbs	13	-29.81	1.30	-4.38	1.82
		Graminoids	42	-29.14#	1.54	2.07	3.16
		Mosses	13	-27.97¤	1.15	-3.37	0.90
		Salix polaris	16	-29.57€	0.93	-4.18	1.14

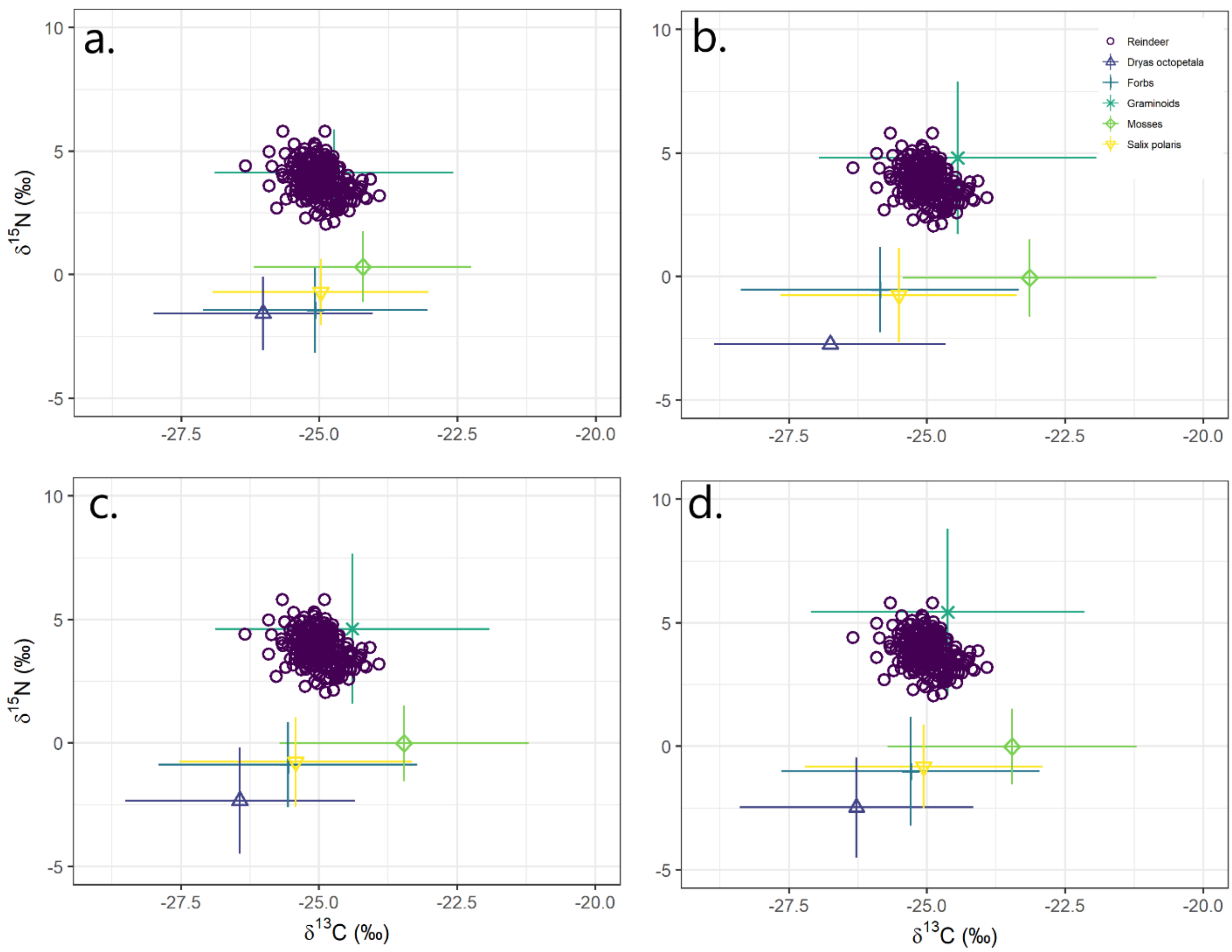

Figure S1: Biplot of Svalbard Reindeer serum $\delta^{13}C$ and $\delta^{15}N$ and their winter food sources that have been corrected for trophic discrimination for the four separate models that were created to evaluate the best source means to utilize. The different models are shown in panels: (a) model 1; (b) model 2; (c) model 3 and (d) model 4. All forage and serum $\delta^{13}C$ values were corrected to 2012 using the method outlined in Schubert & Jahren (2012).

Table S4: Deviance information criterion (DIC) of the different models to determine the most appropriate sources to use in the mixing models to reconstruct the diets of the Svalbard Reindeer The lowest DIC values are in the light grey boxes. The ΔDIC is calculated between models 1 and 4 and is the higher DIC value minus the lower DIC value.

**Year**	**DIC**				**ΔDIC**
	**Model 1**	**Model 2**	**Model 3**	**Model 4**	
1995	86.72	94.94	95.15	92.59	5.87
1996	79.76	81.95	82.40	78.99	0.77
1997	84.68	87.90	88.47	85.22	0.54
1998	99.50	102.11	102.68	98.93	0.57
1999	87.82	86.12	86.43	82.41	5.41
2000	93.87	98.83	99.20	95.92	2.05
2001	68.14	73.17	73.28	70.95	2.81
2002	95.68	101.93	102.51	99.02	3.33
2004	98.67	104.07	104.33	100.69	2.02
2005	112.90	110.46	111.51	106.13	6.77
2006	98.08	99.62	100.65	95.99	2.09
2007	108.32	105.64	106.46	101.05	7.27
2008	124.59	120.34	121.50	114.61	9.98
2009	111.60	109.47	110.60	105.20	6.40
2011	130.82	126.53	127.87	120.98	9.84
2012	165.87	158.54	160.03	151.38	14.49

1.3 Stable isotope mixing model (simmr) outputs

Standard practice in evaluating data and its suitability to be modelled in mixing models requires that once the food sources have been corrected for trophic enrichment (discrimination) all the consumer's data must fall within the mixing polygon derived from all sources (Phillips et al., 2014). The current data set was considered suitable for running in the simmr package (Versions 0.4.5) in R as all the data points fall within the mixing polygon (Figure S2).

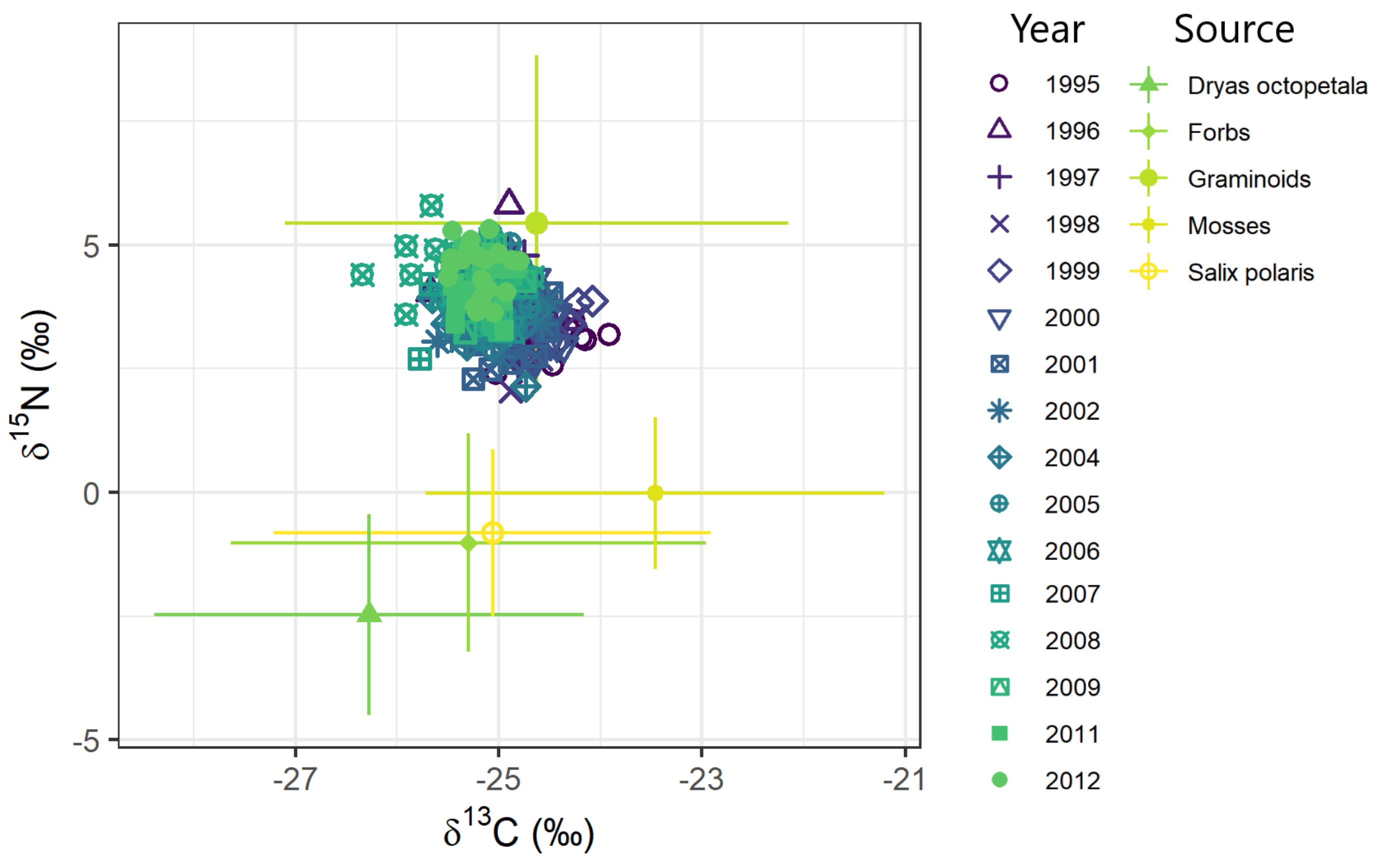

Figure S2: Biplot of Svalbard Reindeer serum $\delta^{13}C$ and $\delta^{15}N$ and their winter food sources (4=W_9_13_19) that have been corrected for trophic discrimination. All forage and serum $\delta^{13}C$ values were corrected to 2012 using the method outlined in Schubert & Jahren (2012).

1.3.1 Model diagnostics

Table S5: Brooks, Gelman, Rubin (BGR) convergence diagnostics for each source and year modelled in the stable isotope mixing model (simmr). The BGR test was used to check for convergence and to evaluate the fitting performance of the models and the values should all be close to 1. All the years modelled showed convergence and good model fit.

Year	deviance	*Dryas octopetala*	Forbs	Graminoids	Mosses	*Salix polaris*	*sd*[δ^{13}C]	*sd*[δ^{15}N]
**1995**	1	1	1	1	1	1	1	1
**1996**	1	1	1	1	1	1	1	1
**1997**	1	1	1	1	1	1	1	1
**1998**	1	1	1	1	1	1	1	1
**1999**	1	1	1	1	1	1	1	1
**2000**	1	1	1	1	1	1	1	1
**2001**	1	1	1	1	1	1	1	1
**2002**	1	1	1	1	1	1	1	1
**2004**	1	1	1	1	1	1	1	1
**2005**	1	1	1	1	1	1	1	1
**2006**	1	1	1	1	1	1	1	1
**2007**	1	1	1	1	1	1	1	1
**2008**	1	1	1	1	1	1	1	1
**2009**	1	1	1	1	1	1	1	1
**2011**	1	1	1	1	1	1	1	1
**2012**	1	1	1	1	1	1	1	1

Table S6: Proportion of observations (y) that lie outside the posterior predictive (**yrep**) of 50% each year modelled with late winter priors in the stable isotope mixing model (simmr). The highest proportion of observations that lie outside **yrep** is 0.467 in 2008, less than the 0.5 specified in the posterior predictive function. For all other years, the proportion of observations that lie outside **yrep** are in the range 0.133 to 0.400, indicating all the models fit well.

Year	Proportion outside posterior predictive interval of 50%
**1995**	0.133
**1996**	0.400
**1997**	0.154
**1998**	0.200
**1999**	0.292
**2000**	0.200
**2001**	0.136
**2002**	0.200
**2004**	0.267
**2005**	0.200
**2006**	0.250
**2007**	0.321
**2008**	0.467
**2009**	0.300
**2011**	0.294
**2012**	0.310

1.3.2 Matrix plots

The matrix plots containing the posterior probability distributions show unimodal and narrow peaks indicating the models were able to differentiate between the five sources (Figures S3-S6). Correlations among the sources in the matrix plots can suggest a lot about the model uncertainty and possibly help determine the best fitting models in terms of the priors utilized. Very large negative correlations indicate that the model cannot separate the two sources as they are close in isospace, while large positive correlations indicate there are multiple competing sources that are contributing to the mixture (Phillips et al., 2014). The negative correlations between graminoids and mosses which are not close in isospace, range between -0.45 and -0.63. Graminoids and *Salix polaris* are also not close in isospace have negative correlations ranging from -0.30 and -0.61. Mosses and *Salix polaris* overlap in isospace, and the negative correlations range from -0.07 to -0.48. These correlations are considered not to be very large, thus indicating the models are able to discern the contributions of the different sources to the mix.

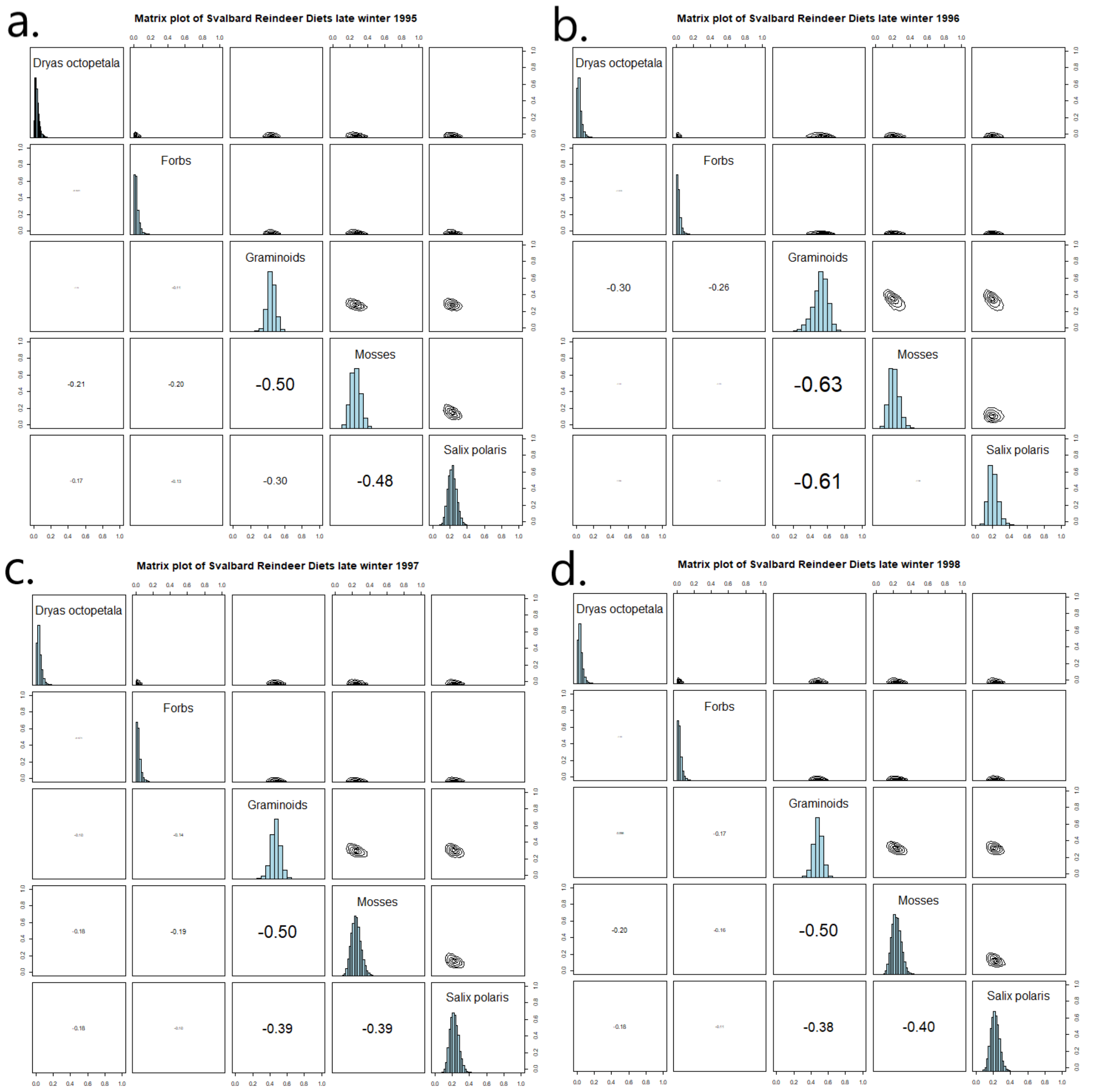

Figure S3: Matrix plot of food sources for the Svalbard Reindeer during the late winter foraging periods of 1995 (a), 1996 (b), 1997 (c) and 1998 (d). The diagonal cells show the posterior probability distributions for each of the food sources (*Dryas octopetala*, Forbs, Graminoids, Mosses and *Salix polaris*). The cells below the diagonal show the correlations between contributions for pairs of food sources. The cells above the diagonal show contours of the joint posterior probability distribution for contributions for pairs of food sources.

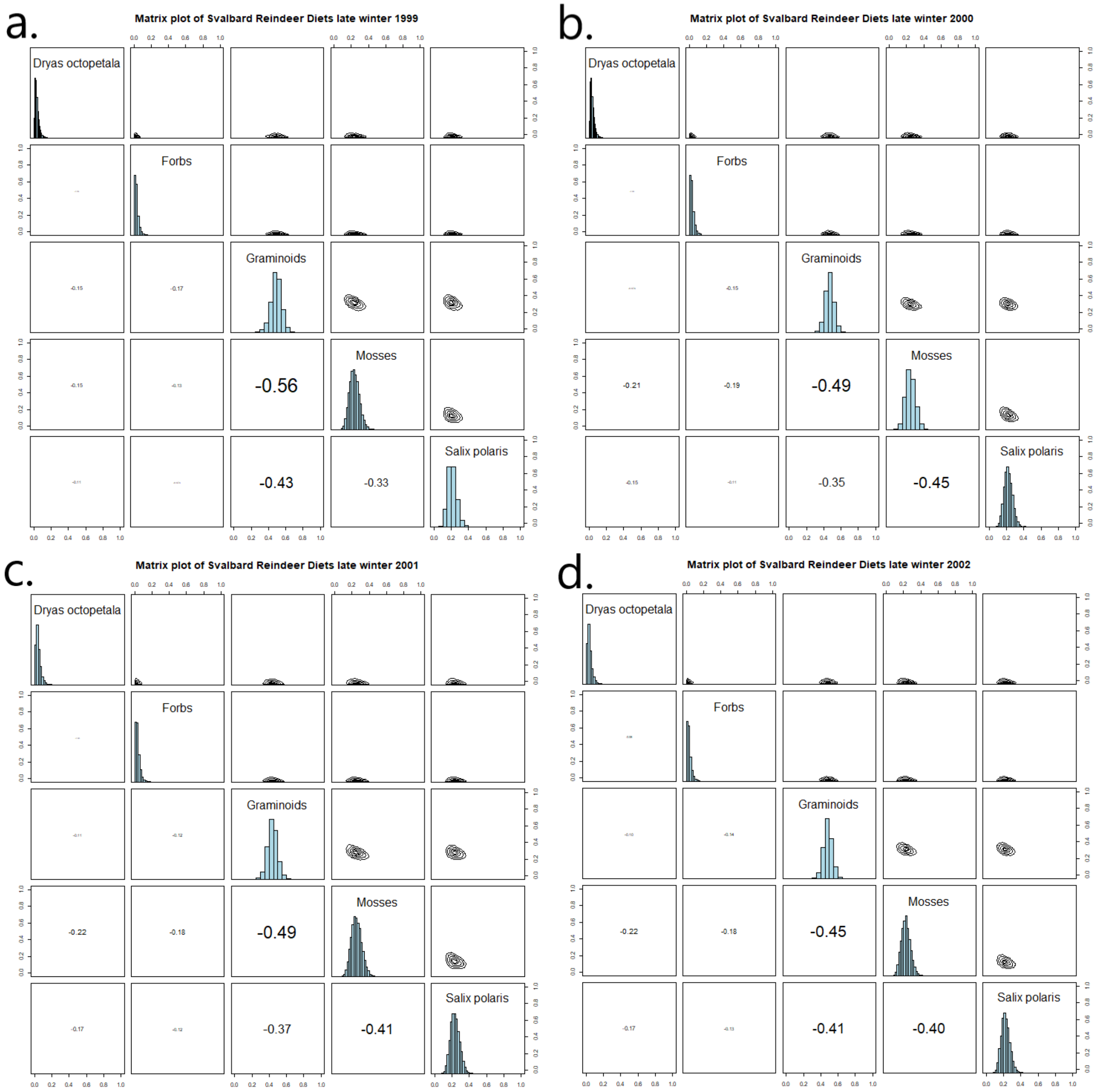

Figure S4: Matrix plot of food sources for the Svalbard Reindeer during the late winter foraging periods of 1999 (a), 2000 (b), 2001 (c) and 2002 (d). The diagonal cells show the posterior probability distributions for each of the food sources (*Dryas octopetala*, Forbs, Graminoids, Mosses and *Salix polaris*). The cells below the diagonal show the correlations between contributions for pairs of food sources. The cells above the diagonal show contours of the joint posterior probability distribution for contributions for pairs of food sources.

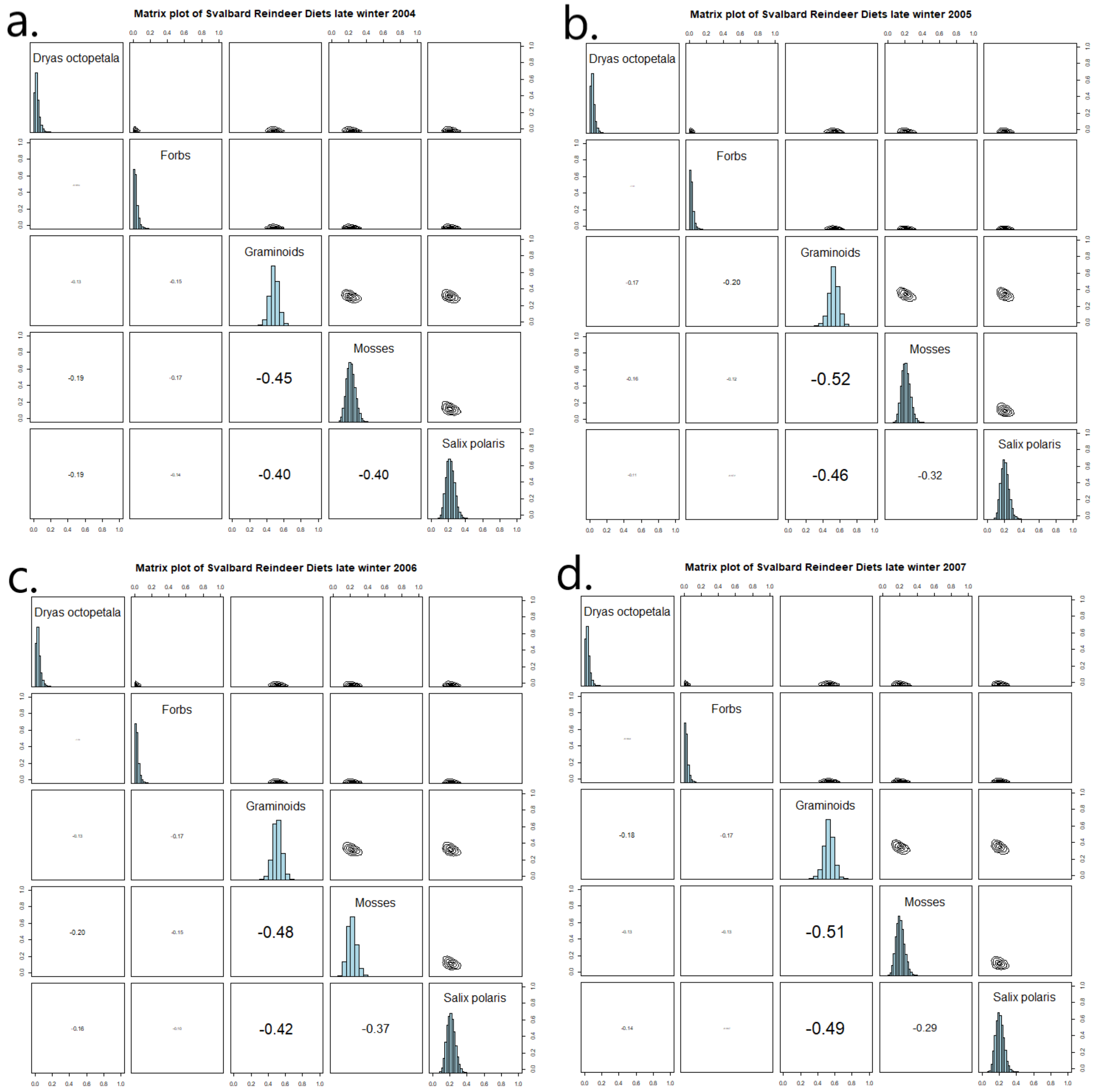

Figure S5: Matrix plot of food sources for the Svalbard Reindeer during the late winter foraging periods of 2004 (a), 2005 (b), 2006 (c) and 2007 (d). The diagonal cells show the posterior probability distributions for each of the food sources (*Dryas octopetala*, Forbs, Graminoids, Mosses and *Salix polaris*). The cells below the diagonal show the correlations between contributions for pairs of food sources. The cells above the diagonal show contours of the joint posterior probability distribution for contributions for pairs of food sources.

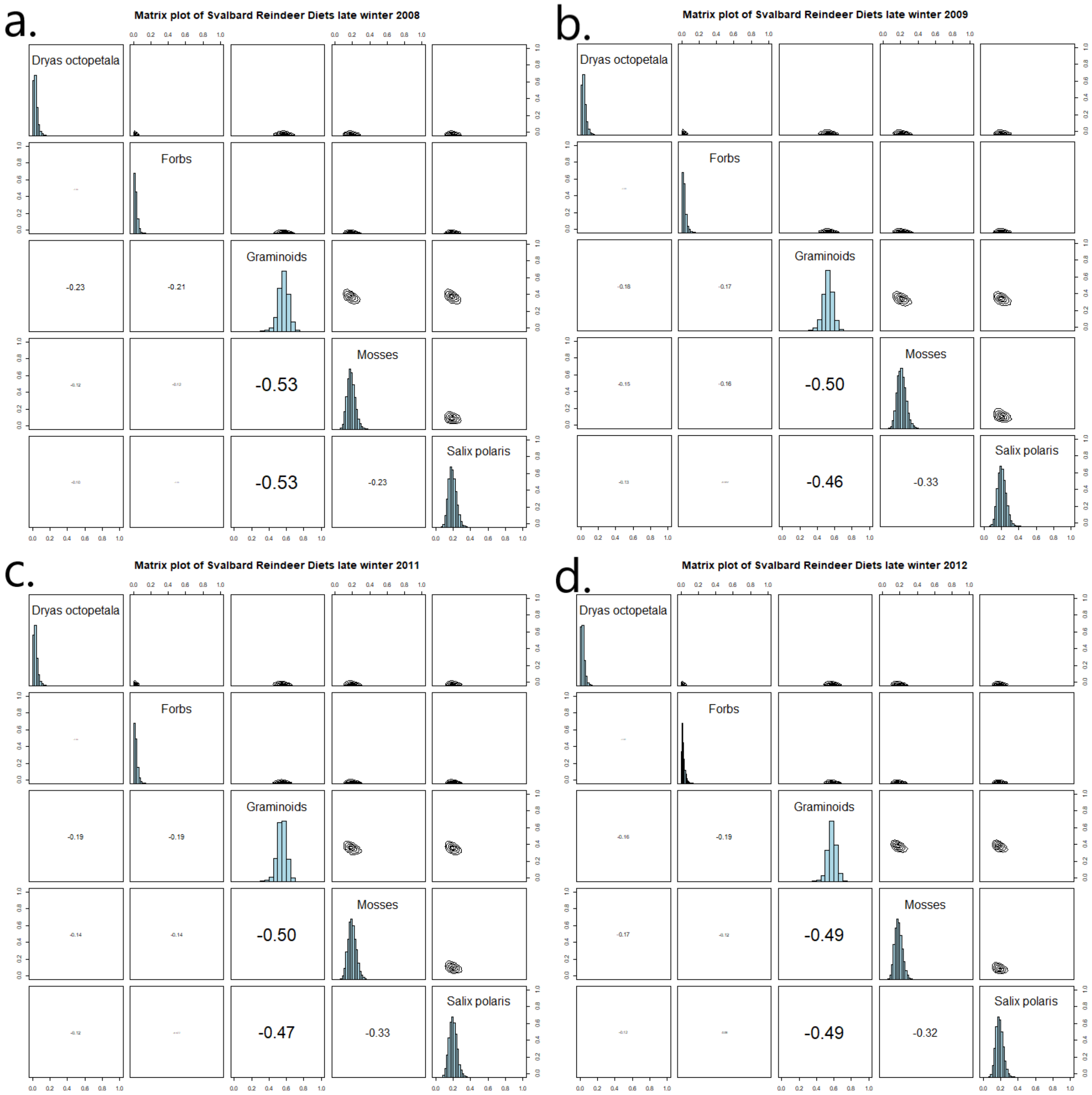

Figure S6: Matrix plot of food sources for the Svalbard Reindeer during the late winter foraging periods of 2008 (a), 2009 (b), 2011 (c) and 2012 (d). The diagonal cells show the posterior probability distributions for each of the food sources (*Dryas octopetala*, Forbs, Graminoids, Mosses and *Salix polaris*). The cells below the diagonal show the correlations between contributions for pairs of food sources. The cells above the diagonal show contours of the joint posterior probability distribution for contributions for pairs of food sources.

1.3.3 Dietary proportion estimates: credible intervals

Table S7: Summary quantiles from the posterior distributions for each of the five sources (*Dryas octopetala*, Forbs, Graminoids, Mosses and *Salix polaris*) and years modelled in the simmr package in R to reconstruct the diets of Svalbard Reindeer from the carbon ($\delta^{13}C$) and nitrogen ($\delta^{15}N$) isotopic values. Serum was collected from female Svalbard reindeer (n=232) in the Reindalen valley system, Nordenskiöldland, Spitsbergen between 1995 and 2012 (excluding 2003 and 2010).

	Quantiles	1995	1996	1997	1998	1999	2000	2001	2002	2004	2005	2006	2007	2008	2009	2011	2012
	2.50%	85.323	68.210	77.661	91.455	74.399	88.670	63.711	91.485	93.007	97.472	88.009	92.337	104.749	96.700	112.452	142.471
	25%	86.657	70.172	78.932	92.752	75.773	89.929	65.051	92.862	94.440	98.828	89.379	93.815	106.313	98.080	113.814	143.896
**deviance**	50%	87.951	72.211	80.319	94.125	77.310	91.259	66.369	94.190	95.851	100.368	90.764	95.306	107.991	99.536	115.345	145.421
	75%	89.954	74.896	82.365	96.179	79.514	93.298	68.379	96.249	97.903	102.727	92.901	97.634	110.444	101.829	117.648	147.704
	97.50%	95.918	81.264	88.512	102.181	85.417	99.237	74.181	102.328	103.809	109.142	99.089	104.243	117.581	108.329	124.285	154.500
	2.50%	0.007	0.006	0.008	0.007	0.007	0.007	0.008	0.007	0.007	0.007	0.007	0.007	0.006	0.007	0.007	0.006
	25%	0.019	0.017	0.019	0.019	0.017	0.019	0.021	0.020	0.020	0.018	0.019	0.018	0.017	0.018	0.017	0.016
Dryas octopetala	50%	0.031	0.029	0.031	0.031	0.028	0.030	0.034	0.032	0.033	0.029	0.031	0.030	0.027	0.029	0.028	0.025
	75%	0.049	0.046	0.050	0.049	0.045	0.048	0.055	0.051	0.052	0.045	0.048	0.047	0.043	0.046	0.044	0.040
	97.50%	0.100	0.109	0.107	0.105	0.098	0.099	0.120	0.108	0.112	0.096	0.104	0.101	0.097	0.099	0.093	0.083
	2.50%	0.006	0.005	0.005	0.006	0.005	0.006	0.006	0.005	0.005	0.005	0.005	0.005	0.005	0.005	0.005	0.005
	25%	0.015	0.013	0.015	0.015	0.014	0.015	0.016	0.015	0.015	0.013	0.014	0.014	0.012	0.014	0.013	0.013
**Forbs**	50%	0.026	0.022	0.025	0.025	0.023	0.025	0.026	0.025	0.025	0.022	0.024	0.023	0.020	0.023	0.022	0.021
	75%	0.043	0.036	0.041	0.041	0.038	0.041	0.044	0.042	0.042	0.037	0.039	0.037	0.034	0.037	0.035	0.034
	97.50%	0.099	0.092	0.100	0.096	0.091	0.096	0.102	0.097	0.102	0.086	0.092	0.085	0.077	0.091	0.082	0.079
	2.50%	0.352	0.329	0.360	0.376	0.360	0.373	0.334	0.387	0.388	0.406	0.401	0.408	0.442	0.407	0.441	0.477
	25%	0.412	0.470	0.433	0.447	0.453	0.436	0.403	0.450	0.453	0.494	0.471	0.495	0.532	0.490	0.516	0.547
**Graminoids**	50%	0.441	0.526	0.467	0.482	0.492	0.468	0.438	0.482	0.486	0.529	0.505	0.530	0.569	0.525	0.549	0.578
	75%	0.473	0.576	0.502	0.515	0.531	0.500	0.475	0.515	0.519	0.563	0.540	0.567	0.606	0.559	0.582	0.608
	97.50%	0.538	0.660	0.571	0.582	0.609	0.565	0.553	0.582	0.588	0.630	0.610	0.638	0.676	0.629	0.645	0.668
	2.50%	0.157	0.115	0.141	0.134	0.133	0.145	0.144	0.132	0.126	0.118	0.123	0.114	0.104	0.120	0.112	0.106
	25%	0.221	0.171	0.202	0.194	0.195	0.207	0.209	0.190	0.184	0.171	0.179	0.167	0.152	0.173	0.163	0.151
**Mosses**	50%	0.260	0.208	0.239	0.230	0.232	0.243	0.248	0.224	0.218	0.205	0.214	0.199	0.181	0.206	0.193	0.179
	75%	0.300	0.249	0.277	0.267	0.274	0.283	0.291	0.259	0.255	0.240	0.249	0.234	0.214	0.242	0.226	0.211
	97.50%	0.379	0.339	0.359	0.342	0.356	0.358	0.375	0.335	0.330	0.315	0.324	0.310	0.286	0.318	0.294	0.274
	2.50%	0.137	0.114	0.133	0.130	0.123	0.131	0.140	0.133	0.133	0.121	0.126	0.120	0.112	0.120	0.117	0.111
	25%	0.190	0.166	0.186	0.184	0.174	0.185	0.196	0.186	0.186	0.169	0.177	0.170	0.158	0.169	0.165	0.154
Salix polaris	50%	0.223	0.200	0.219	0.216	0.206	0.216	0.230	0.217	0.218	0.199	0.209	0.201	0.186	0.200	0.194	0.181
	75%	0.257	0.238	0.254	0.249	0.241	0.250	0.268	0.251	0.253	0.232	0.243	0.235	0.218	0.233	0.225	0.211
	97.50%	0.327	0.326	0.328	0.318	0.317	0.320	0.345	0.324	0.325	0.301	0.313	0.305	0.284	0.303	0.292	0.272
	2.50%	0.012	0.017	0.014	0.011	0.014	0.012	0.015	0.014	0.014	0.015	0.014	0.015	0.018	0.016	0.014	0.013
	25%	0.120	0.173	0.135	0.128	0.138	0.123	0.149	0.135	0.144	0.139	0.141	0.151	0.175	0.143	0.131	0.125
**sd[δ^{13}C]**	50%	0.259	0.375	0.286	0.269	0.298	0.258	0.314	0.282	0.302	0.296	0.304	0.329	0.373	0.294	0.282	0.264
	75%	0.451	0.658	0.501	0.463	0.522	0.452	0.559	0.500	0.521	0.512	0.528	0.573	0.642	0.508	0.491	0.453
	97.50%	0.931	1.465	1.060	0.985	1.130	0.951	1.229	1.063	1.093	1.071	1.158	1.224	1.296	1.080	1.020	0.923
	2.50%	0.020	0.064	0.024	0.022	0.032	0.023	0.022	0.022	0.022	0.027	0.029	0.032	0.035	0.029	0.026	0.027
	25%	0.197	0.614	0.247	0.248	0.314	0.223	0.241	0.218	0.224	0.282	0.253	0.296	0.332	0.285	0.262	0.271
**sd[δ^{15}N]**	50%	0.416	1.273	0.538	0.533	0.671	0.470	0.517	0.462	0.488	0.621	0.544	0.641	0.695	0.609	0.575	0.564
	75%	0.722	2.129	0.933	0.929	1.183	0.819	0.899	0.814	0.848	1.107	0.958	1.113	1.197	1.060	1.027	0.970
	97.50%	1.516	4.275	1.988	1.930	2.507	1.753	1.963	1.743	1.781	2.334	2.051	2.375	2.563	2.284	2.161	2.015

2. Isotopic niche widths & overlaps modelled in SIBER

The data and R scripts that support the findings are openly available in Dryad Digital Repository at https://doi.org/10.5061/dryad.ghx3ffbs7	
Data:	Reindeer_data_schubert_correction.csv
Rcode	R_Code-SIBER_SvalbardReindeer.R

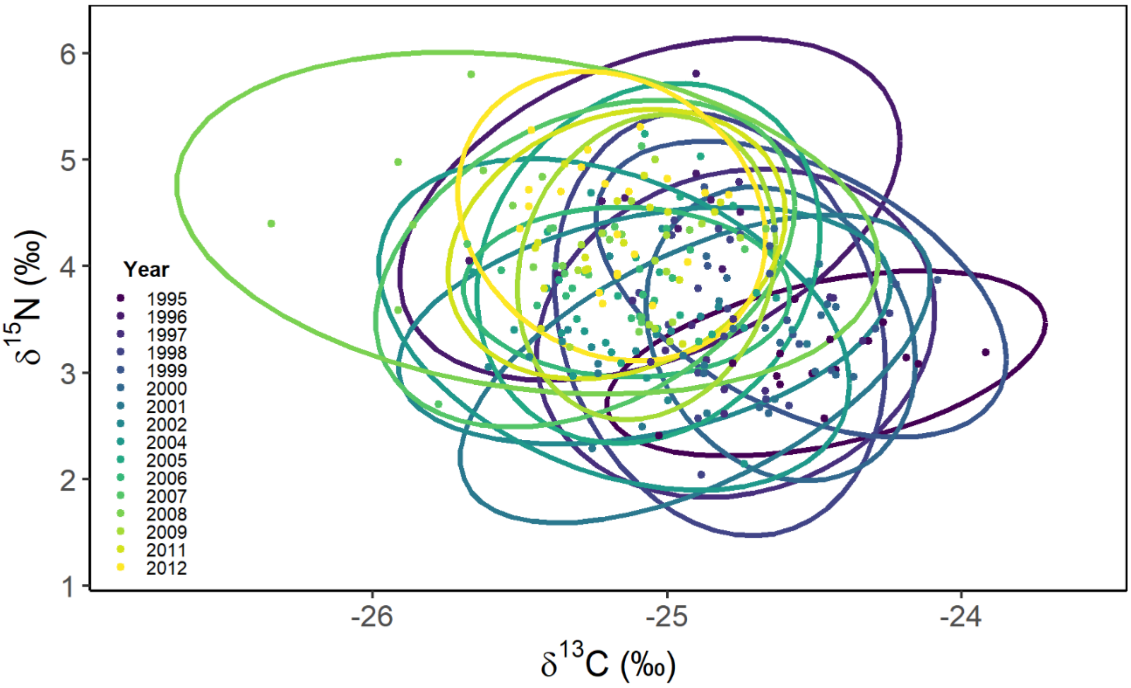

Figure S7: Bivariate plots of serum δ^{13}C and δ^{15}N showing the SIBER standard ellipse areas surrounding the reconstructed isotopic niche spaces and 95% CIs of female Svalbard reindeer (n=232) from the Reindalen valley system, Nordenskiöldland, Spitsbergen between 1995 and 2012 (excluding 2003 and 2010). The SEAs for the individual years were corrected for small sample sizes (SEA_C).

2.1 Summary Statistics

Summary statistics for each year include the following: convex hull total area (TA) and the standard ellipse area corrected for small sample size corrected (SEA_C) (Table S8). To further exclude variability in the sample sizes Bayesian multivariate normal distributions are fitted to each group in the dataset and the SEA_B for each group was calculated (Figure S7).

For posterior comparisons, we tested the probability of one group's SEA_B being bigger than the other group by comparing the proportion of posterior ellipses (PP) that differed between groups. Relevant differences in SEA_B are expected to be reflected by a PP ≥ 0.95, every year's isotopic niche areas differ in size significantly from at least one other year (Table S8). The overlap between ellipses of the different years has been calculated in Table S8. The proportion of overlap varies between the different years from 0% (e.g.1995 & 2012) to 71% (e.g.2011 & 2012).

Table S8: SIBER summary area statistics of the reconstructed isotopic niches using δ13C and δ15N values of female Svalbard Reindeer serum (n=232) from the Reindalen valley system, Nordenskiöldland, Spitsbergen between 1995 and 2012 (excluding 2003 and 2010). Summary statistics include convex hull total area (TA), standard ellipse area corrected for small sample size (SEA_C) and the Bayesian Standard ellipse area (SEA_B). The bold text highlights the pairs of years at the beginning (1995;1996) and end (2011;2012) of the study where the first year had no or little ROS and the second year had extreme ROS (>60mm).

**Year**	**1995**	**1996**	**1997**	**1998**	**1999**	**2000**	**2001**	**2002**	**2004**	**2005**	**2006**	**2007**	**2008**	**2009**	**2011**	**2012**
**TA**	**0.66**	**0.91**	0.93	1.06	0.64	0.61	0.71	1.00	1.41	1.00	0.42	1.26	2.31	0.63	**0.82**	**0.84**
**SEA_C**	**0.26**	**0.52**	0.46	0.50	0.37	0.28	0.40	0.40	0.53	0.44	0.20	0.46	0.84	0.28	**0.33**	**0.33**
**SEA_B**	**0.24**	**0.43**	0.40	0.44	0.32	0.24	0.35	0.35	0.47	0.39	0.17	0.41	0.74	0.24	**0.30**	**0.30**

Regardless of the increased proportion of graminoids in the diet *per se* (especially years 2001–2012), there is high variance in the isotopic niche space (SEA_B, Figure S8). This variability could firstly originate from interannual differences in forage biomass production (van der Wal & Stien, 2014) and secondly from the varying isotopic values of different graminoid species (Matthews & Mazumder, 2004; Yeakel et al., 2016). The functional group of graminoids is composed of grasses (*Poaceae*), sedges (*Cyperaceae*), and rushes (*Juncaceae*). These three families encompass species that are important in the diets of Svalbard reindeer, such as species of *Alopecurus*, *Dupontia*, *Poa*, *Deschampsia* and *Festuca* (grasses), *Eriophorum* (a sedge) and *Luzula* (a rush) (Bjørkvoll et al., 2009; Staaland, 1984; Zhao et al., 2019). When plant availability was limited by ROS in the winters of 2001 and 2002, Svalbard reindeer diets were found to consist mainly of erect graminoid species from mesic sites (*Alopecurus*, *Poa*, and *Festuca*) or rushes (*Luzula)* and dwarf shrubs (*S. polaris*) (Bjørkvoll et al., 2009). These different graminoids vary in their quality and have different and variable isotopic signatures. For example, the mean isotopic values of the high quality, preferred *Alopecurus alpina* were -28.32±1.3 ($\delta^{13}C$) and 4.95±3.8 ($\delta^{15}N$) whereas for the low quality, unfavourable *Luzula confusa* they were -30.65±0.8 ($\delta^{13}C$) and 1.63±3.0 ($\delta^{15}N$) (Staaland, 1984; Zhao et al., 2019). While the consumption of graminoids has increased, the isotopic niche variance may be due to the selection and utilisation of varying combinations of favourable and unfavourable graminoid species under different conditions.

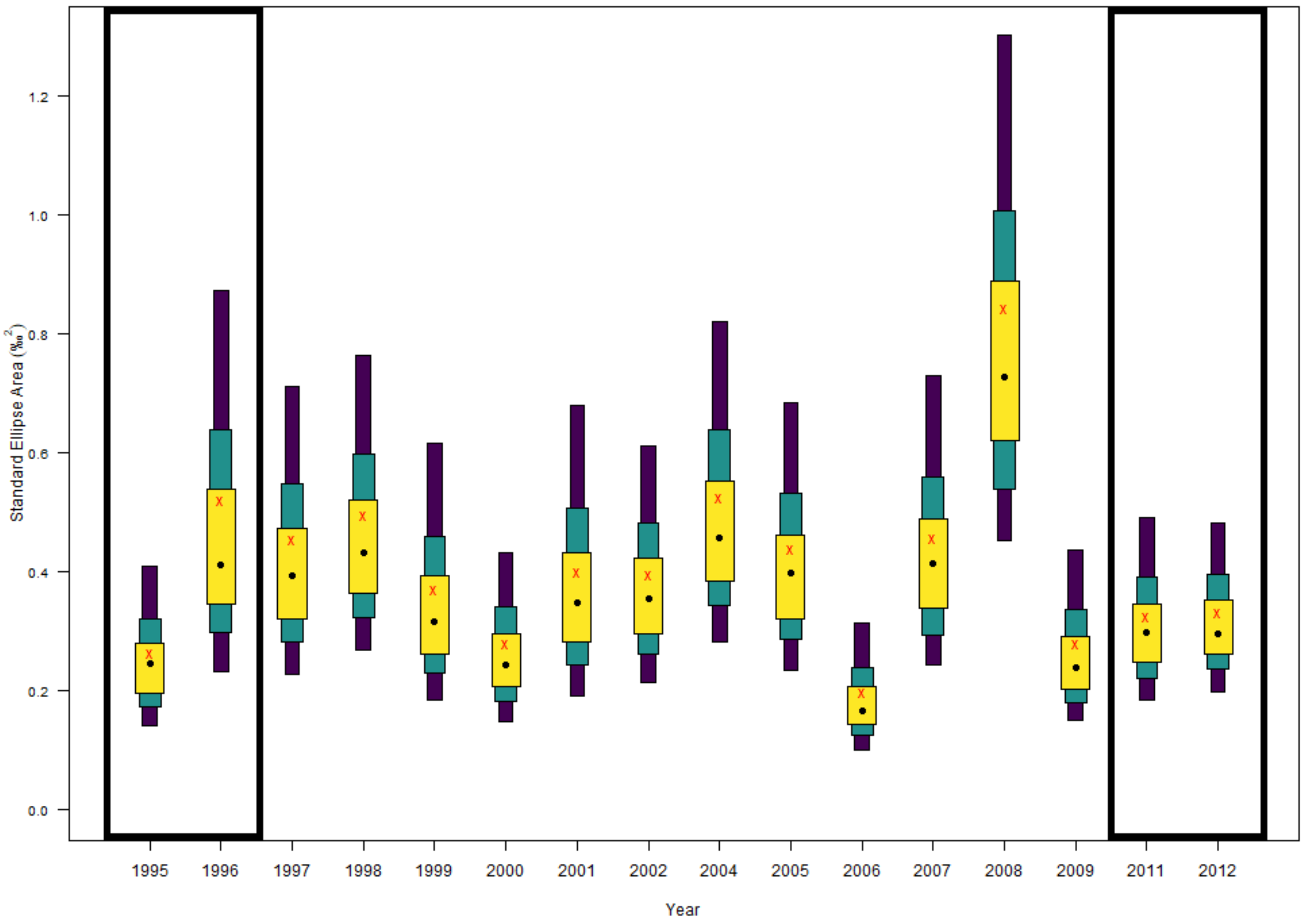

Figure S8: Boxplot of the sizes of the Bayesian standard ellipse areas (SEA_B) modelled SIBER using the serum $\delta^{13}C$ and $\delta^{15}N$ values. Black dots represent the mode, red crosses represent the mean while the boxes represent 50%, 75%, and 95% credible intervals. The black boxes highlight the pairs of years at the beginning (1995;1996) and end (2011;2012) of the study where the first year had no or little ROS and the second year had extreme ROS (>60mm). See Parnell et al., (2013) for more information on isotopic niche modelling and the associated metrics.

Table S9: Probability that isotopic niche area of female Svalbard Reindeer of years in group A are bigger than the years in group B. Isotopic niches were constructed using $\delta^{13}C$ and $\delta^{15}N$ values of reindeer serum (n=232) from the Reindalen valley system, Nordenskiöldland, Spitsbergen between 1995 and 2012 (excluding 2003 and 2010). Values with a PP ≥ 0.95 (yellow highlight) indicates year A is significantly bigger than B while values with a PP ≤ 0.05 (green highlight) indicates that year A is significantly smaller than B. The bold text highlights the pairs of years at the beginning (1995;1996) and end (2011; 2012) of the study where the first year had no or little ROS and the second year had extreme ROS (>60mm).

		B															
	Year	1995	1996	1997	1998	1999	2000	2001	2002	2004	2005	2006	2007	2008	2009	2011	2012
A	1995		**0.053**	0.084	0.047	0.192	0.442	0.143	0.139	0.032	0.087	0.785	0.073	0.002	0.449	**0.272**	**0.244**
	1996			0.611	0.525	0.770	0.931	0.696	0.729	0.465	0.643	0.988	0.592	0.115	0.931	**0.869**	**0.866**
	1997				0.398	0.677	0.885	0.593	0.626	0.336	0.518	0.980	0.463	0.052	0.891	0.804	0.799
	1998					0.763	0.935	0.692	0.722	0.433	0.625	0.992	0.570	0.081	0.935	0.872	0.873
	1999						0.764	0.416	0.431	0.189	0.338	0.947	0.288	0.023	0.767	0.624	0.601
	2000							0.182	0.172	0.046	0.116	0.824	0.094	0.002	0.509	0.328	0.298
	2001								0.520	0.264	0.418	0.962	0.374	0.040	0.824	0.712	0.692
	2002									0.225	0.398	0.965	0.342	0.021	0.828	0.703	0.686
	2004										0.694	0.994	0.637	0.107	0.955	0.908	0.904
	2005											0.981	0.444	0.040	0.888	0.790	0.780
	2006												0.015	0.000	0.179	0.083	0.067
	2007													0.059	0.907	0.826	0.824
	2008														0.998	0.996	0.996
	2009															0.323	0.292
	2011																**0.476**
	2012																

Table S10: Comparison of isotopic ellipse overlaps of Svalbard Reindeer serum $\delta^{13}C$ and $\delta^{15}N$ between all years. Isotopic niches were constructed using $\delta^{13}C$ and $\delta^{15}N$ values of reindeer serum (n=232) from the Reindalen valley system, Nordenskiöldland, Spitsbergen between 1995 and 2012 (excluding 2003 and 2010). The bold text highlights the pairs of years at the beginning (1995; 1996) and end (2011; 2012) of the study where the first year had no or little ROS and the second year had extreme ROS (>60mm).

Year	1995	1996	1997	1998	1999	2000	2001	2002	2004	2005	2006	2007	2008	2009	2011	2012
1995		**0%**	37%	28%	36%	43%	33%	18%	19%	9%	5%	1%	4%	6%	**0%**	**0%**
1996			21%	30%	22%	12%	11%	27%	34%	55%	32%	56%	45%	41%	**61%**	**57%**
1997				69%	48%	58%	61%	49%	45%	38%	27%	22%	22%	33%	22%	19%
1998					41%	48%	47%	43%	43%	47%	26%	30%	29%	43%	32%	28%
1999						49%	29%	28%	18%	27%	17%	17%	20%	23%	20%	18%
2000							44%	34%	24%	21%	17%	10%	14%	17%	11%	10%
2001								47%	43%	28%	20%	15%	15%	21%	12%	10%
2002									56%	49%	44%	41%	29%	40%	32%	24%
2004										47%	38%	53%	35%	37%	39%	31%
2005											44%	61%	43%	64%	64%	53%
2006												38%	23%	53%	50%	39%
2007													48%	53%	71%	58%
2008														31%	39%	39%
2009															64%	56%
2011																**71%**
2012																

**Legend**
0%
1-25%
26-50%
51-75%
76-99%
100%

3. Linear Mixed Effects Models

The data and R scripts that support the findings are openly available in Dryad Digital Repository at https://doi.org/10.5061/dryad.ghx3ffbs7	
Data:	Reindeer_data_schubert_correction.csv
Rcode	R_Code-GLMMs_SvalbardReindeer.R

3.1 Fixed Factors

3.1.1 Body Mass of Samples Svalbard Reindeer through the study period

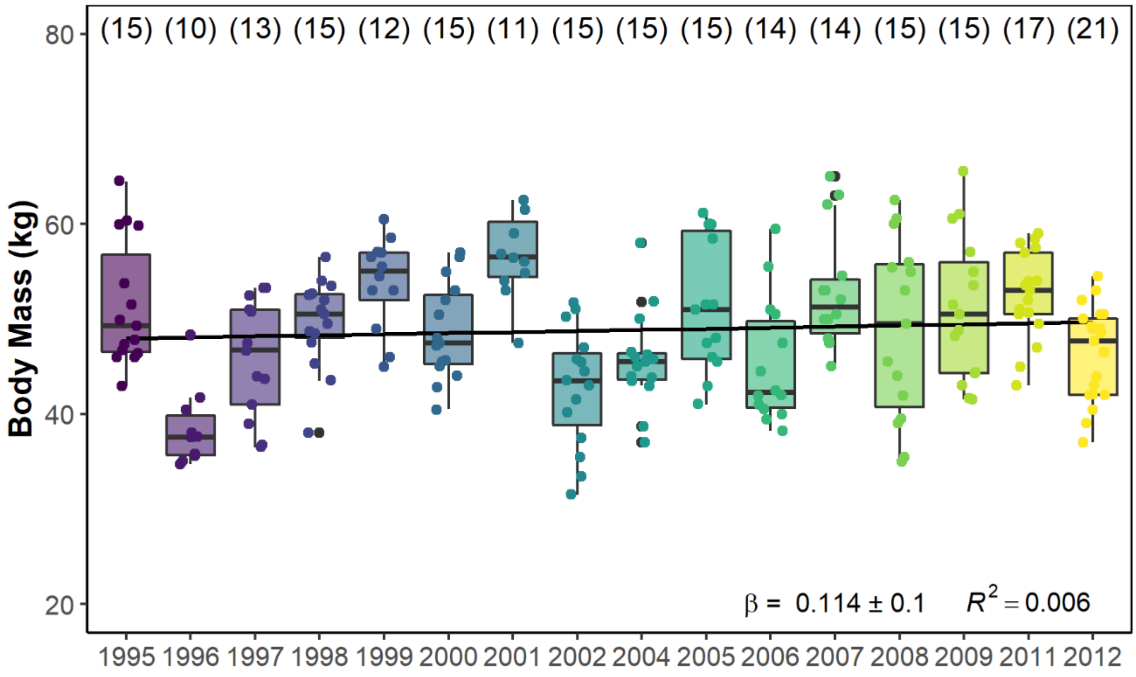

Figure S9: Body mass of female Svalbard reindeer (n=232) captured and weighed in the Reindalen valley system, Nordenskiöldland, Spitsbergen between 1995 and 2012 (excluding 2003 and 2010). Box and whisker plots showing body mass values (median, 25%-75%, interquartile range, nonoutlier range and outliers [black points]). Individual observations are represented by the points. Sample sizes of the reindeer are indicated within brackets.

3.1.2 Svalbard Reindeer Population in Reindalen

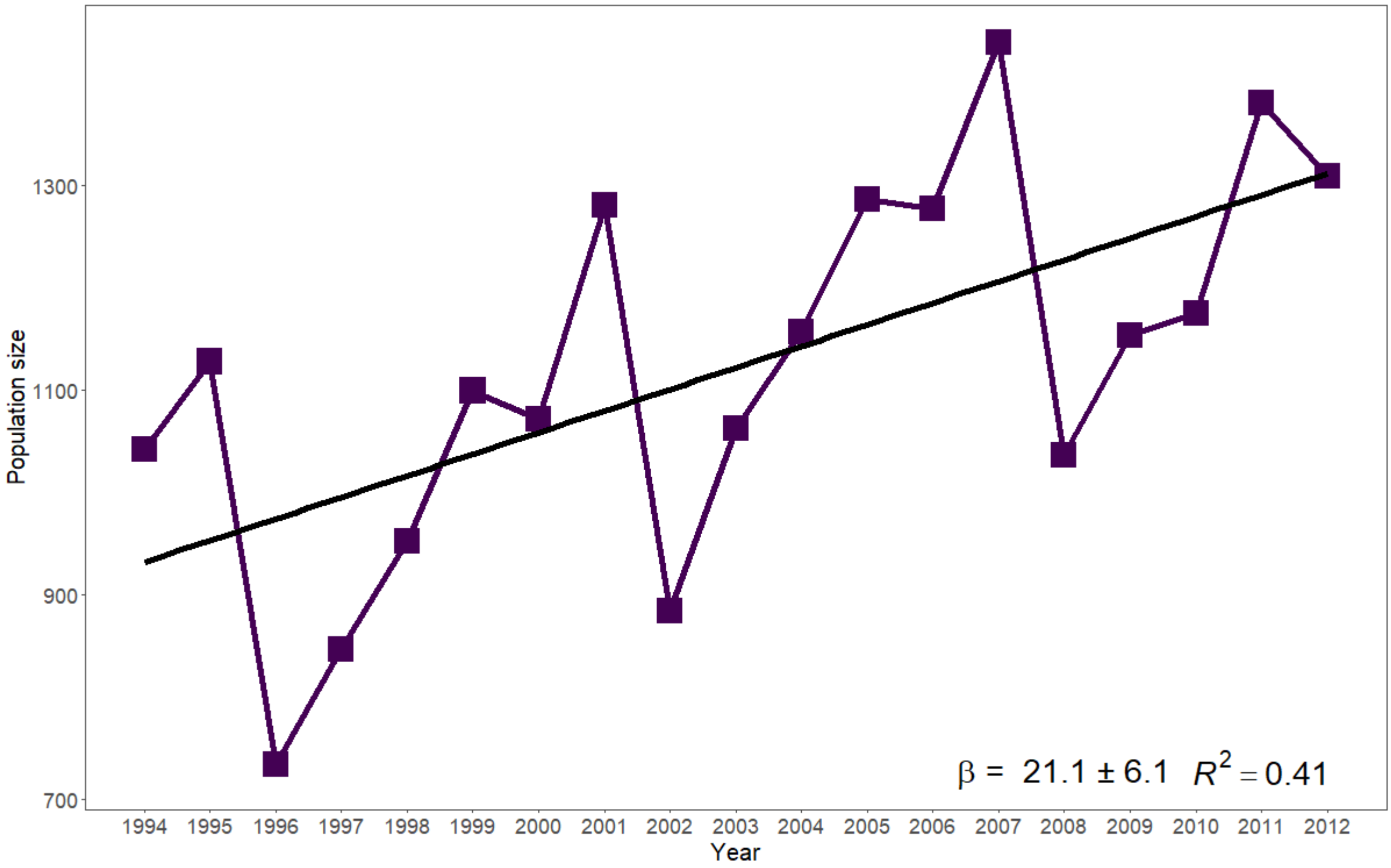

Figure S10: Total yearly Svalbard Reindeer population size estimates (females and claves of both sexes) in Reindalen from 1994 to 2012 with fitted linear regression (black line; Albon et al., 2017).

3.1.3 Rain-on-Snow

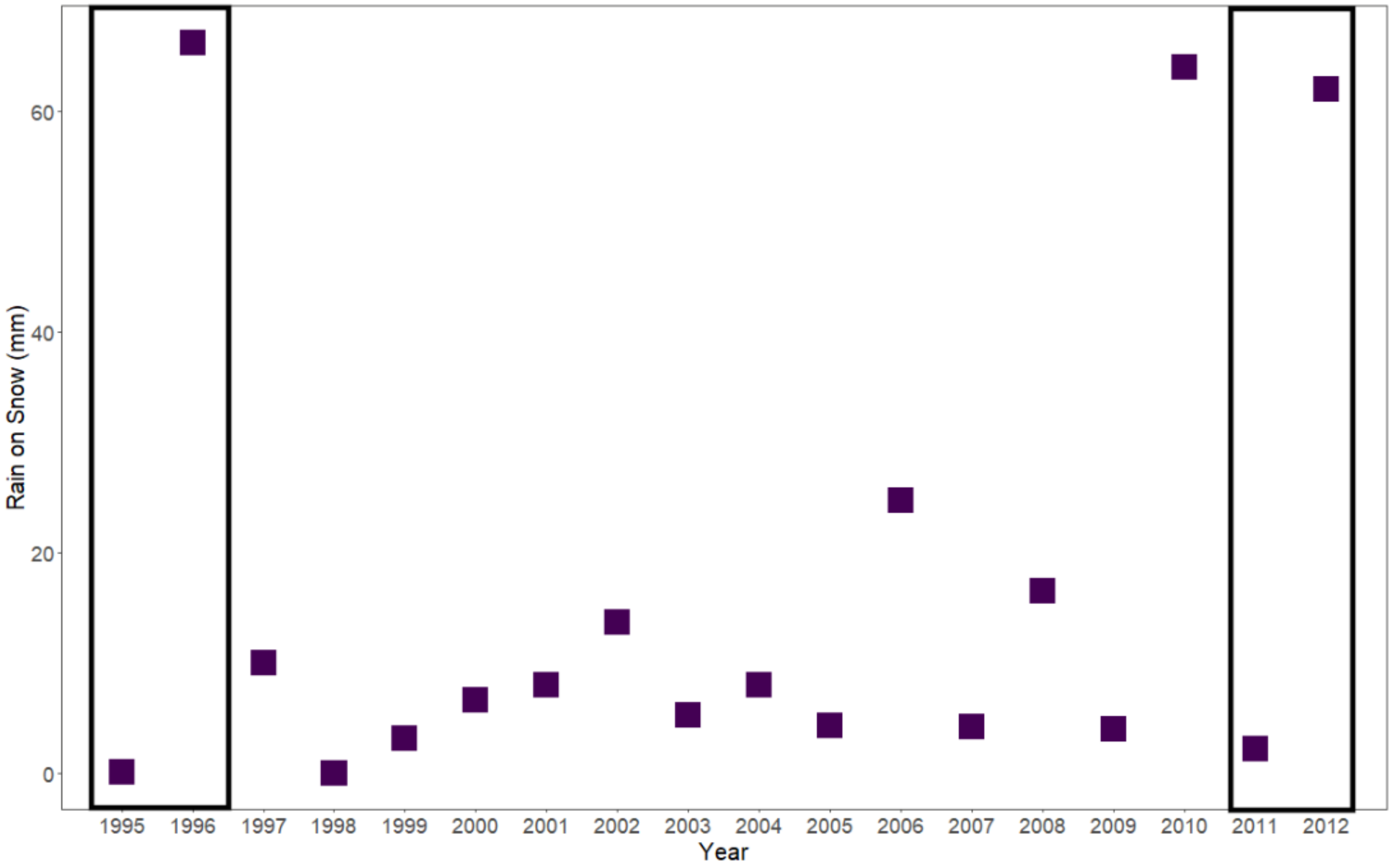

Figure S11: Total yearly rain-on-snow (ROS) for the winters prior to capture and sampling of Svalbard Reindeer from 1995 to 2012. Data obtained from Peeters et al. (2019). The black boxes highlight the pairs of years at the beginning (1995;1996) and end (2011;2012) of the study where the first year had no or little ROS and the second year had extreme ROS (>60mm).

Extreme ROS events greater than 60 mm occurred three times in the study period (Figure S11).

- 1996: Total ROS = 66.2mm in two large scale rainfall events in December 1995 and March 1996
- 2010: Total ROS = 64mm in one large scale rainfall event in January 2010.
- 2012: Total ROS = 62mmin one large scale rainfall event in January 2012 followed by two smaller events in February and March.

3.1.4 Temperature

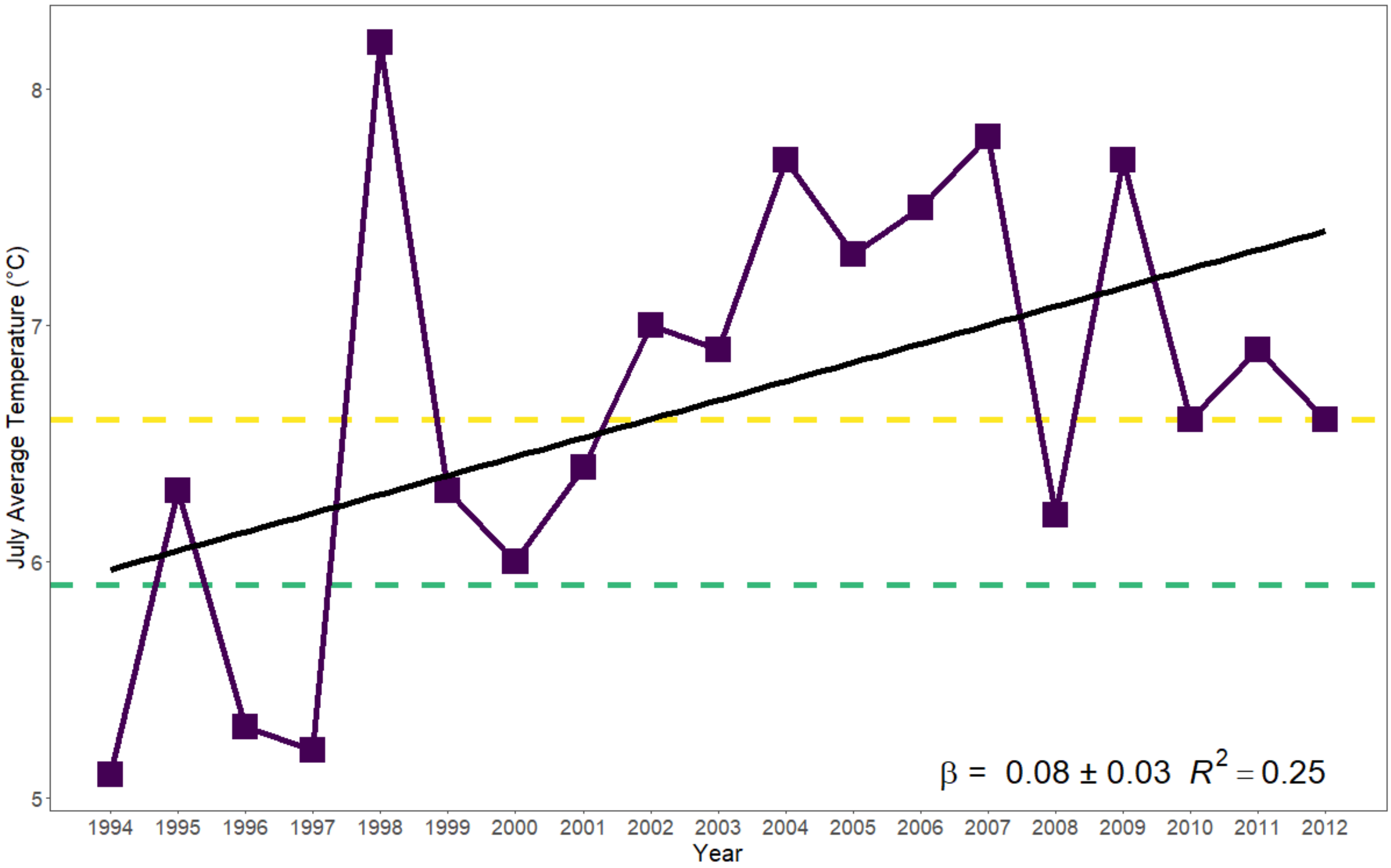

Figure S12: July Average temperatures obtained from Svalbard Airport for the period 1994 to 2012 for the summer prior to capture and sampling of Svalbard Reindeer with fitted linear regression. The dashed green line represents the long term at July average temperature for the period 1961 – 1990 (5.9 °C) and the dashed yellow line represents the July average temperature (6.6 °C) for the study period (1994 -2012). Data obtained from the obtained were from the Norwegian Meteorological Institute (2021).

3.2 Model Selection

Table S11: Candidate models assessing the variation in $\delta^{13}C$ and $\delta^{15}N$ values of female Svalbard reindeer (n=232) sampled between 1995 and 2012 (excluding 2003 and 2010). Intrinsic predictors were body mass and pregnancy (only $\delta^{15}N$), while extrinsic predictors were rain-on-snow (ROS), July average temperature, and population density. ROS and population density were log-transformed in the $\delta^{13}C$ analysis only. Female identity (ID) and year were included as random intercept effects. The models were fitted with the default Restricted Maximum Likelihood (REML) and the number of degrees of freedom (K), the conditional Akaike Information Criterion (cAIC) and the difference in cAIC (ΔcAIC) are presented. In addition, the estimated proportion of variance explained (R^2) by the fixed factors alone (marginal R^2, $R^2_{LMM(m)}$) and by both the fixed and random factors (conditional R^2; $R^2_{LMM(c)}$) are presented. The best models (i.e. a ΔcAIC <2) are in black text while the full (selected) models are highlighted in bold text The correlation between year and average $\delta^{13}C$ and $\delta^{15}N$ values in the raw data ($\delta^{13}C$: r = -.78; $\delta^{15}N$: r = .58) was reduced when the $\delta^{13}C$ and $\delta^{15}N$ levels were measured by the annual residual values, corrected for estimated fixed effects ($\delta^{13}C$: r = -.41; $\delta^{15}N$: r = .32). The fixed effect predictor variables in the selected models do not predict all the variance in the temporal trends in $\delta^{13}C$ and $\delta^{15}N$.

		Model	K	cAIC	ΔcAIC	$R^2_{GLMM(m)}$ (%)	$R^2_{GLMM(c)}$ (%)
$\delta^{13}C$	*Intrinsic*	*Body Mass + (1 | Year) + (1 | ID)*	*48.41*	*42.69*	*2.14*	1.62%	61.72%
	Intrinsic and Extrinsic	ROS + Body Mass + (1 | Year) + (1 | ID)	53.65	40.55	0.00	15.74%	62.51%
		ROS + July Average Temperature + Body Mass + (1 | Year) + (1 | ID)	50.49	42.02	1.47	20.13%	63.10%
		**ROS + July Average Temperature + Population + Body Mass+ (1 | Year) + (1 | ID)**	**48.85**	**42.30**	**1.75**	**26.73%**	**63.42%**
		Population + Body Mass + (1 | Year) + (1 | ID)	44.70	43.28	2.73	24.05%	60.97%
		July Average Temperature + Body Mass + (1 | Year) + (1 | ID)	45.12	44.01	3.46	11.63%	61.87%
$\delta^{15}N$	*Intrinsic*	*Pregnancy + Body Mass+(1 | Year) + (1 | ID)*	*113.01*	*273.95*	*2.08*	7.70%	78.38%
	Intrinsic and Extrinsic	Population + Pregnancy + Body Mass+(1 | Year) + (1 | ID)	114.38	271.87	0.00	21.34%	78.18%
		**ROS + July Average Temperature + Population + Pregnancy + Body Mass + (1 | Year) + (1 | ID)**	**113.48**	**272.52**	**0.65**	**34.31%**	**77.74%**
		ROS + Pregnancy + Body Mass + (1 | Year) + (1 | ID)	113.42	272.60	0.73	24.32%	77.25%
		ROS + July Average Temperature + Pregnancy + Body Mass + (1 | Year) + (1 | ID)	112.62	273.41	1.54	34.10%	77.10%
		July Average Temperature + Pregnancy + Body Mass + (1 | Year) + (1 | ID)	112.49	274.82	2.95	18.07%	78.13%

4. References

Albon, S. D., Irvine, R. J., Halvorsen, O., Langvatn, R., Loe, L. E., Ropstad, E., Veiberg, V., van der Wal, R., Bjørkvoll, E. M., Duff, E. I., Hansen, B. B., Lee, A. M., Tveraa, T., & Stien, A. (2017). Contrasting effects of summer and winter warming on body mass explain population dynamics in a food-limited Arctic herbivore. *Global Change Biology*, *23*(4), 1374–1389. https://doi.org/10.1111/gcb.13435

Ben-David, M., Shochat, E., & Adams, L. G. (2001). Utility of stable isotopes analysis in studying foraging ecology of herbivores: examples from moose and caribou. *Alces*, *37*(2), 421–434. https://alcesjournal.org/index.php/alces/article/view/527

Bjørkvoll, E., Pedersen, B., Hytteborn, H., Jónsdóttir, I. S., & Langvatn, R. (2009). Seasonal and Interannual Dietary Variation During Winter in Female Svalbard Reindeer (Rangifer Tarandus Platyrhynchus). *Arctic, Antarctic, and Alpine Research*, *41*(1), 88–96. https://doi.org/10.1657/1523-0430-41.1.88

Hansen, B. B., Lorentzen, J. R., Welker, J. M., Varpe, Ø., Aanes, R., Beumer, L. T., & Pedersen, Å. Ø. (2019). Reindeer turning maritime: Ice-locked tundra triggers changes in dietary niche utilization. *Ecosphere*, *10*(4), e02672. https://doi.org/10.1002/ecs2.2672

Matthews, B., & Mazumder, A. (2004). A critical evaluation of intrapopulation variation of δ13C and isotopic evidence of individual specialization. *Oecologia*, *140*(2), 361–371. https://doi.org/10.1007/s00442-004-1579-2

MOSJ. (2021). *Seasonal temperatures for Svalbard Airport. Environmental monitoring of Svalbard and Jan Mayen (MOSJ).* http://www.mosj.no/en/climate/atmosphere/temperature-precipitation.html

Parnell, A. C., Phillips, D. L., Bearhop, S., Semmens, B. X., Ward, E. J., Moore, J. W., Jackson, A. L., Grey, J., Kelly, D. J., & Inger, R. (2013). Bayesian stable isotope mixing models. *Environmetrics*, *24*(6), 387–399. https://doi.org/10.1002/env.2221

Peeters, B., Pedersen, Å. Ø., Loe, L. E., Isaksen, K., Veiberg, V., Stien, A., Kohler, J., Gallet, J.-C., Aanes, R., & Hansen, B. B. (2019). Spatiotemporal patterns of rain-on-snow and basal ice in high Arctic Svalbard: detection of a climate-cryosphere regime shift. *Environmental Research Letters*, *14*(1), 015002. https://doi.org/10.1088/1748-9326/aaefb3

Phillips, D. L., Inger, R., Bearhop, S., Jackson, A. L., Moore, J. W., Parnell, A. C., Semmens, B. X., & Ward, E. J. (2014). Best practices for use of stable isotope mixing models in food-web studies. *Canadian Journal of Zoology*, *92*, 823–835. https://doi.org/10.1139/cjz-2014-0127

Schubert, B. A., & Jahren, A. H. (2012). The effect of atmospheric CO 2 concentration on carbon isotope

fractionation in C 3 land plants. *Geochimica et Cosmochimica Acta*, *96*, 29–43. https://doi.org/10.1016/j.gca.2012.08.003

Staaland, H. (1984). On the quality of Svalbard reindeer pasture in the summer and autumn. *Rangifer*, *4*(1), 16. https://doi.org/10.7557/2.4.1.487

van der Wal, R., & Stien, A. (2014). High-arctic plants like it hot: A long-term investigation of between-year variability in plant biomass. *Ecology*, *95*(12), 3414–3427. https://doi.org/10.1890/14-0533.1.sm

Yeakel, J. D., Bhat, U., Elliott Smith, E. A., & Newsome, S. D. (2016). Exploring the isotopic niche: Isotopic variance, physiological incorporation, and the temporal dynamics of foraging. *Frontiers in Ecology and Evolution*, *4*(JAN), 1–16. https://doi.org/10.3389/fevo.2016.00001

Zhao, L. Z., Colman, A. S., Irvine, R. J., Karlsen, S. R., Olack, G., & Hobbie, E. A. (2019). Isotope ecology detects fine-scale variation in Svalbard reindeer diet: implications for monitoring herbivory in the changing Arctic. *Polar Biology*, *42*(4), 793–805. https://doi.org/10.1007/s00300-019-02474-8
